# Beyond the app: a marketness assessment of digital health, sports, and well-being - a systematic review

**DOI:** 10.3389/fdgth.2026.1871114

**Published:** 2026-10-08

**Authors:** Francisco von Hafe, Ana Rita Santos, Ana Rita Londral, Ana Margarida Silva, José Lopes, Nuno Carvalho, Soraia Ferreira, Salomé Azevedo

**Affiliations:** 1 Value for Health CoLAB, Lisboa, Portugal; 2 Comprehensive Health Research Center, Faculdade de Ciências Médicas, Universidade NOVA de Lisboa, Lisboa, Portugal; 3 Nova School of Business and Economics, Universidade NOVA de Lisboa, Carcavelos, Portugal; 4 Nova National School of Public Health, Universidade Nova de Lisboa, Lisbon, Portugal; 5 Uppsala Health Economics, Department of Public Health and Caring Sciences, Uppsala University, Uppsala, Sweden; 6 Department of Physics, Nova School of Science and Technology, Universidade NOVA de Lisboa, Lisbon, Portugal; 7 Department of Product Design, Dotmoovs, Braga, Portugal; 8 Department of Machine Learning, Dotmoovs, Braga, Portugal; 9 Universidade de Évora, Escola de Saúde e Desenvolvimento Humano, Departamento de Desporto e Saúde, Évora, Portugal; 10 Sport, Performance & Exercise Applied Research (SPEAR LAB), Polytechnic University of Portalegre, Portalegre, Portugal; 11 Biosciences Higher School of Elvas, Polytechnic University of Portalegre, Portalegre, Portugal; 12 CEG-IST, Instituto Superior Técnico, Universidade de Lisboa, Lisboa, Portugal

**Keywords:** digital adoption, digital health, digital sports, digital well-being, health economics, market dynamics, marketness, systematic review

## Abstract

**Background:**

Digital health, sports, and well-being solutions have proliferated rapidly, yet most fail to sustain continued use, even when effective. This gap cannot be explained solely by solution-level factors; it also reflects the economic settings in which they operate. In settings that function as markets, prices, competition, and user choice create incentives that can sustain participation over time. Marketness, the degree to which a setting displays the characteristics of a market, provides a diagnostic framework to assess what the existing evidence does and does not reveal. Without characterizing marketness, investment, regulation, and participation decisions are made without a clear understanding of the market conditions that shape sustained participation.

**Methods:**

Following PRISMA guidelines, we systematically searched PubMed, Web of Science, and EconLit for empirical studies of digital health, sports, or well-being solutions. Included studies were assessed against Rosenbaum's four marketness criteria: (1) voluntary and specified exchange, (2) typified goods or services, (3) regularity of exchange, and (4) competition. Studies were grouped into value proposition categories, and each category was then positioned along the marketness continuum, from hierarchy (non-market) to fully constituted market, based on the evidence synthesized across the studies within that category.

**Results:**

Seventeen studies were grouped into three value proposition categories: condition management (*n* = 6), lifestyle and behavior (*n* = 8), and physical activity (*n* = 3). No category met all four marketness criteria. Condition management approaches the hierarchy end: participation is sustained by clinical endorsement and institutional validation, not market mechanisms. Lifestyle and behavior approaches the market end: exchange terms are commercial, participation is freely initiated, but logging burden, motivational decay, and privacy concerns disrupt regularity. Critically, no included study examined the competition criterion, agent switching, price pressure, or competitive dynamics.

**Conclusion:**

The sustained participation gap points to a broader empirical gap: these settings have rarely been examined as markets. The economic mechanisms that could, in principle, sustain participation, competition, pricing, and supply-side responses remain empirically uncharacterized. Future research should examine participation as a comparative choice among alternatives, estimate demand-side parameters from freemium and subscription data, and study developer pricing and competitive responses, providing the empirical foundation currently missing from investment decisions, regulatory frameworks, and participation strategies.

## 1 Introduction

Physical inactivity and chronic diseases affect approximately one-third of adults worldwide and are major contributors to the global burden of disease, driving morbidity, mortality, and rising healthcare costs (1–3). In response, digital solutions in health, sports, and well-being have emerged as scalable approaches to support disease prevention, behavior change, and self-management (4). Digital health refers to solutions enabling individuals to manage their health, access care, and improve health-related outcomes through digital means (5); digital sports refers to solutions that enable or enhance participation in sport and physical activity through digital technologies, including e-sports and active exergames, while supporting performance monitoring and improvement (6); and digital well-being refers to solutions supporting individuals in achieving their mental, emotional, and lifestyle goals (7). Despite their rapid proliferation, with more than 350,000 solutions available and 3.6 billion downloads in 2024, most fail to sustain use over time, with usage typically dropping between 20% to 30% within the first month (8, 9), even when effectiveness has been demonstrated (10). This gap between market scale and sustained usage limits solutions’ potential impact and raises concerns about inefficient resource investment (11, 12). Part of this gap can be attributed to solution-level factors such as usability, design, and content quality (13). Content quality has been identified as a facilitator of engagement independent of usability barriers (14), while content tailored to users’ goals has been associated with increased engagement in mobile Health (mHealth) solutions (15). However, sustained participation also depends on the decisions of the agents who interact with these solutions, and those decisions are shaped by the broader economic setting in which both the solutions and the agents operate (16).

Those decisions are shaped by a range of considerations: each agent chooses whether to adopt a solution and how intensively to use it, weighing expected benefits against monetary costs (subscription fees, device requirements, data charges) and non-monetary costs (time, cognitive effort, privacy risks, and the opportunity cost associated with alternative activities) (17–19). Expected benefits are themselves conditional on the quality and relevance of the content or service delivered (13–15): a solution that is easy to use but offers little engaging or valuable content may provide the user with no intrinsic or extrinsic reason to adopt it in the first place, regardless of how low its usability costs are. Content quality therefore operates plays a prior role in the cost-benefit calculation described above, not merely alongside it. This weighing of expected benefits against costs is also shaped by the agent's readiness to change, a concept describing the different stages agents move through before adopting a new behavior, from not yet intending to act to sustaining the change over time (20). Evidence from stage-based models applied to nutrition and fitness apps shows that potential users vary widely in this readiness, ranging from those who have decided to adopt but have not yet started to those who tried a solution before and have since stopped (21). Readiness to change therefore introduces a source of variation in participation that is external to the solution itself: identical prices, features, and content can yield different adoption and continuation outcomes purely as a function of where different individuals stand in this process.

In many digital contexts, moreover, these decisions are also influenced by the choices of other agents, as digital solutions frequently operate as platforms connecting distinct users whose participation decisions are mutually interdependent: the value each user derives depends on the participation of the others (22, 23). This interdependence creates cross-side network effects, whereby participation by one group influences the benefits derived by another. Because participation decisions depend not only on a solution's direct benefits and costs but also on the behavior of other agents, they must be understood within the broader economic setting in which these interactions occur, that is, the market in which agents and solutions participate. This market shapes the constraints agents face, the alternatives available to them, and the incentives to which they respond (24). In digital contexts, however, markets are particularly difficult to delineate, as the boundaries between exchange, platform intermediation, and ecosystem participation are often blurred (25).

Despite being central to economic analysis, the concept of “market” lacks a single universally accepted definition (26). Rosenbaum identifies three broad categories of definitions: observational, focusing on empirical phenomena such as the exchange of specific goods; functional, focusing on what markets do, such as price formation and resource allocation; and structural, focusing on underlying networks of stable exchange relationships (24). Given these conceptual ambiguities, marketness offers a useful way to characterize the degree to which a setting displays the characteristics of a market. Rather than treating markets as simply present or absent, marketness conceptualizes them along a continuum from hierarchical non-market forms to fully constituted markets (27). Marketness is proposed to be assessed through four criteria: voluntary and specified exchange, meaning participation is uncoerced and the terms of exchange are defined; typified goods or services, meaning what is exchanged is sufficiently standardized to be recognizable across transactions; regularity, meaning exchange recurs with sufficient consistency to constitute a stable pattern; and competition, meaning multiple buyers or sellers exert pressure on one another (24). This framework has been applied to study other contexts, such as the biodiversity offset markets, which assessed wetland mitigation banking in the United States against the same four criteria. It found that, while exchange is voluntary and the goods are partially typified, regularity is weak, and competition is largely absent, positioning these markets as hybrid forms closer to the hierarchy end of the continuum. The study concluded that this partial marketness has direct policy implications: the analytical tools appropriate for fully constituted markets, such as price-based coordination and competitive regulation, cannot be straightforwardly applied to settings that only partially display market characteristics (28).

To the authors’ knowledge, no equivalent marketness assessment exists for digital health, sports, and well-being settings. As a result, investment decisions, regulatory frameworks, and participation strategies in these contexts are designed without knowing which analytical tools apply or whether the mechanisms they assume are present. This systematic review aims to address that gap. Evidence on how these solutions operate as markets is dispersed across disciplines (29), with studies reporting only partial elements of agents, interactions, exchange conditions, and competitive dynamics, making systematic synthesis necessary (30). Following the structural definition of market as the most amenable to empirical assessment (24), and building on network-based approaches proven well-suited to analyze market structures (31–33), we examine, guided by research questions (Table 1), what solutions are studied; which agents participate and under what terms; what interaction types characterize these settings; and what factors shape agents’ decisions to enter, remain in, or exit. For each setting, we assess the extent to which the available evidence positions it along the marketness continuum, providing the diagnostic foundation needed to identify which analytical tools are applicable and what evidence must be gathered before they can be deployed.

**Table 1:** Research questions.

Research question	Objective
What digital solutions are studied?	To identify typified goods or services, their degree of standardization across transactions, and the pricing structures under which they are exchanged.
Which agents participate in these markets?	To identify agents, their roles, and the terms governing exchange, distinguishing decision-makers with endogenous participation from those who shape the incentive structure faced by others.
What interaction types characterize these markets?	To characterize how agents coordinate, transmit information, and structure exchange, addressing the conditions under which exchange is specified and whether platform intermediation creates cross-side interdependencies.
What factors shape competitive dynamics and agents’ participation over time?	To identify the drivers and frictions governing entry, continued use, and exit, distinguishing monetary and non-monetary cost components, and to assess what these patterns reveal about regularity and the extent to which competitive pressure among solutions shapes participation.

## 2 Materials and methods

This study was structured into three sequential phases. Phase 1 (Section 2.1) details the review design, search strategy, and study selection, culminating in the final corpus of included studies. Phase 2 (Section 2.2) describes the data extraction framework operationalized through the four marketness criteria. Phase 3 (Section 2.3) explains the synthesis of extracted evidence and the systematic assessment of degrees of marketness across digital health, sports, and well-being contexts.

### 2.1 Review design and process

The systematic literature review was conducted in accordance with the Preferred Reporting Items for Systematic Reviews and Meta-analyses (PRISMA) guidelines (34). The completed PRISMA checklist is provided in the Supplementary File A1. The review phase comprised three stages: (i) literature search, (ii) eligibility assessment and study selection, and (iii) quality assessment. This review was not pre-registered.

#### 2.1.1 Literature search

Search terms were developed from the study objectives and research questions and organized into two conceptual blocks. The first block, “socio-technical-economic networks”, reflects the structural definition of markets adopted in this study, which treats markets as networks of stable exchange relationships. This block operationalizes the search for studies describing the dimensions through which Rosenbaum's four marketness criteria can be assessed: the social dimension captures agent roles, voluntary participation, and peer interactions (voluntary and specified exchange; competition); the technical dimension captures solution characteristics and coordination mechanisms (typified goods or services); and the economic dimension captures pricing structures, incentives, and participation patterns (regularity; competition). The second block, “digital health, sports, and well-being”, identifies the contexts to which the structural definition is applied.

These two blocks were searched across PubMed, Web of Science, and EconLit, selected to jointly cover the disciplinary scope of this review: PubMed for health and clinical literature on digital health and well-being solutions, including sports-related research addressing the medical, physiological, and health aspects of sport (35), Web of Science for its indexing of sport science alongside psychology, information systems, and business (36), and EconLit for the economics literature on pricing, market structure, and consumer behavior central to the marketness framework, supported by evidence that EconLit supplements biomedical database coverage in health policy–related reviews (37). An initial search was conducted in PubMed, Web of Science, and EconLit covering studies published between January 1st, 2020 and December 31st, 2024. This search was subsequently updated to extend coverage through June 30th, 2026, following the same databases, search strategy, and eligibility criteria. Records from both searches were restricted to studies written in English. The 2020 lower bound reflects the marked acceleration in digital health adoption, funding, and implementation driven by the COVID-19 pandemic (38, 39). Pre-2020 studies were conducted under substantially different regulatory and reimbursement conditions (40). Controlled vocabulary (e.g., PubMed MeSH terms) and database-specific syntax were applied (Supplementary File A2).

#### 2.1.2 Eligibility assessment and study selection

Retrieved studies were imported into an Artificial Intelligence (AI)-assisted systematic literature review tool (41), which was used solely for citation management (screening, organization, and record tracking). Before initial screening, duplicate and retracted articles were removed. During the screening stage, titles and abstracts were independently assessed by two authors against the predefined inclusion and exclusion criteria (Box 1). When abstracts were unavailable, full texts were retrieved and screened.

Box 1 **Eligibility criteria for screening titles, abstracts, and full-text papers**

**1. Relevance to Study Scope**

 - Inclusion Criteria

  - Studies addressing digital health, digital sports, or digital well-being solutions.

 - Exclusion Criteria

  - Studies focusing exclusively on digital solutions developed for COVID-19 (e.g., contact tracing, quarantine monitoring).

  - Studies evaluating digital solutions intended only for use within hospitals (i.e., not available for routine out-of-hospital use or public download).

  - Studies analyzing gaming without physical activity (e.g., eSports without bodily movement).

  - Studies examining solutions not available on the market for download or public use (e.g., proprietary research prototypes).

**2. Empirical and Analytical Quality**

 - Inclusion Criteria

  - Studies that clearly describe the context in which the digital solution is used.

 - Exclusion Criteria

  - Study protocols, literature reviews, methodological frameworks, or reports of workshops or training activities without original empirical findings.

  - Studies limited to feature lists or basic user descriptions that do not address at least one of the following dimensions: (i) agents and roles; (ii) the digital solutions; (iii) drivers of entry, retention, or exit; or (iv) interactions.

**3. Thematic Fit**

 - Inclusion Criteria

  - Studies that explicitly analyze the structure of these digital markets or provide sufficient detail to characterize at least one of the following dimensions: (i) agents and roles; (ii) the digital solutions involved; (iii) drivers of entry, retention, or exit; or (iv) interaction.

  - Studies examining payment, pricing, or reimbursement models.

  - Studies analyzing user motivations or behavioral factors affecting adoption, continued use, or discontinuation.

 - Exclusion Criteria

  - Studies that do not identify agents or interactions.

  - Studies limited to social media or app review analytics without contextual information on agent roles, solution use, and participation drivers.

  - Studies focusing solely on solution design without examining implementation, use, or impact.

Full-text articles deemed potentially eligible were subsequently reviewed by the same two authors to confirm alignment with all inclusion criteria and with the study's research questions. Any disagreements were resolved through discussion; when consensus could not be reached, a third author was consulted.

#### 2.1.3 Quality assessment

Study quality was assessed using the Mixed Methods Appraisal Tool (MMAT), version 2018 (42), which supports appraisal of qualitative, quantitative, and mixed-methods studies. Two reviewers independently appraised each included study against the MMAT design-specific criteria; appraisals were compared, and discrepancies were resolved through discussion until full agreement was reached. The MMAT first evaluates the clarity of the research questions and the appropriateness of the collected data. It then applies design-specific criteria, with responses categorized as “Yes”, “No”, or “Can't tell”. As the MMAT does not generate an overall score, appraisal results are reported narratively and used to contextualize the strength of the synthesized evidence. No studies were excluded solely based on quality assessment.

### 2.2 Data extraction and analytical structure

Full-text articles were analyzed using a structured literature matrix (43). Extracted descriptive data included publication year, country of the first author, authors’ affiliations, study design, and study aims. The disciplinary composition of author teams was inferred from institutional affiliations to characterize the interdisciplinarity of the study teams (see Supplementary File A3) (29). To support marketness assessment, we additionally coded each study according to digital solution value proposition, primary users, and the type of solution developer. Studies were assigned to a value proposition category based on each solution's primary stated purpose or target outcome, rather than its underlying technology or delivery format (e.g., mobile app). Two reviewers independently assigned each study to a category; assignments were compared, and disagreements were resolved through discussion, with a third author consulted where consensus could not be reached. Categories represent inductively developed analytical groupings based on shared value propositions.

Solution developers were classified according to organizational anchoring as: (i) private-sector developers; (ii) public or non-profit developers (including academic, hospital, or public agencies); and (iii) cross-sector collaborations, where development involved formal partnerships between private and public or academic organizations (44–48). To contextualize whether each included study aimed to understand an existing market or to evaluate a specific intervention, we additionally coded each study according to its relationship to the solution studied, distinguishing between market studies (used here to denote studies of existing, publicly available solutions rather than studies that necessarily assessed market structure) and solution evaluation studies (in which a specific solution was developed or deployed in an academic or clinical context and evaluated as part of the study).

Data extraction was structured around the four marketness criteria, with each analytical component designed to capture the evidence needed to assess a specific criterion. One reviewer performed data extraction, which a second reviewer independently verified in full against the source articles, with discrepancies resolved by discussion. Inductive codes for variables without predefined categories were developed independently by both reviewers using a grounded theory approach, then discussed and consolidated into a final codebook applied across all included studies. Deductive coding for variables with established classifications used existing peer-reviewed taxonomies, following the same extraction-and-verification process already described. The full extraction framework is reported in Supplementary File A4.

#### 2.2.1 Typified goods or services: digital solutions

Digital solutions were characterized in terms of what is exchanged and how it is standardized across transactions, drawing on the World Health Organization (WHO) Classification of Digital Health Interventions, the Behavioral Intervention Technology (BIT) model (48, 49), and inductively coded behavior-change and persuasive features. Each solution's access and pricing model was additionally coded, as these structures determine the terms under which exchange occurs.

#### 2.2.2 Voluntary and specified exchange: agents and roles

Agents identified in each study were classified by functional role and economic position in the exchange structure, distinguishing agents whose participation is endogenous, shaped by their own expected utility and costs, from agents who shape the incentive structure faced by others. Seven agent categories were coded: primary agents, secondary agents, professional agents, solution developers, distribution platforms, institutional bodies, and peer and community networks (44, 45). For each agent type, the terms of exchange were retrieved: whether participation was voluntary and freely initiated or constrained by clinical protocols, referral pathways, or institutional mandates; and whether exchange terms were freely negotiated, defined by app onboarding, or governed by clinical or regulatory authority.

#### 2.2.3 Regularity and competition: incentives, costs, pricing, and participation decisions

Participation drivers shaping decisions to enter, remain in, or stop using the digital solution were first coded inductively and subsequently consolidated into five categories: endorsement and legitimacy, perceived value and usefulness, pricing and financial incentives, supportive design features, and frictions and burdens (18, 50–54). Perceived value and usefulness encompassed content-related drivers, including content quality, relevance, and effectiveness, alongside broader perceptions of a solution's usefulness to the participant. The framework was additionally explicitly coded for evidence of competitive dynamics, agent switching, price pressure among competing solutions, and developer pricing responses, given their centrality to Rosenbaum's competition criterion.

#### 2.2.4 Interactions and coordination mechanisms

Interactions described in each study were recorded to document how agents coordinate and exert pressure on one another. Interactions were first classified according to whether they occurred between agents (agent–agent) or between agents and digital solutions (agent–solution) (55), and then coded using Thompson's typology of ties, authority/power, instrumental, communication, boundary-penetration, and transaction (56), to capture the coordination mechanisms operating within each digital context and to assess the conditions under which exchange is structured and specified.

### 2.3 Synthesis and marketness assessment

The extracted evidence was synthesized by aggregating the findings reported across the value proposition categories. Evidence gaps were identified by documenting dimensions absent or underreported across studies.

The synthesis then assessed the degree to which each category constitutes a market, evaluated against Rosenbaum's four criteria of marketness. Following the approach of Vaissière and Levrel, each criterion was assessed by describing what the included studies report and positioning the observed evidence along the continuum from hierarchy to a fully constituted market. At this market end, voluntary exchange, typified goods, regularity, and competition are all clearly present and agents coordinate through prices and incentives. In hierarchical or non-market forms, the hierarchy ends, where these criteria are absent or subordinated to authority, governance, or coercion, and participation is determined by mandate rather than choice (28).

Positioning was determined qualitatively. For each category, two authors independently assessed the evidence reported across the included studies against each of Rosenbaum's four criteria and proposed a relative position: hierarchy-leaning, mixed/intermediate, or market-leaning. Disagreements were resolved through discussion, and the resulting positioning was reviewed, refined, and validated by the full author team to ensure analytical consistency. Each criterion was assessed and is reported separately. Evidence gaps were recorded explicitly rather than assigned a default position.

The assessment explicitly distinguishes between two sources of evidence gaps: those reflecting a genuine absence of market features in the settings studied, and those reflecting the absence of research on dimensions that may exist in the settings but have not been examined. This distinction determines whether gaps are substantive findings about market structure or methodological observations about research orientation. The assessment closes with an overall positioning of each category on the marketness continuum.

## 3 Results

The database search initially identified 7,892 records. After duplicate removal and screening at the title/abstract and full-text levels, studies were excluded for lacking relevant data, for the absence of empirical findings, or for the unavailability of the solution in the market, resulting in 17 studies included in the final review (Figure 1).

**Figure 1:**
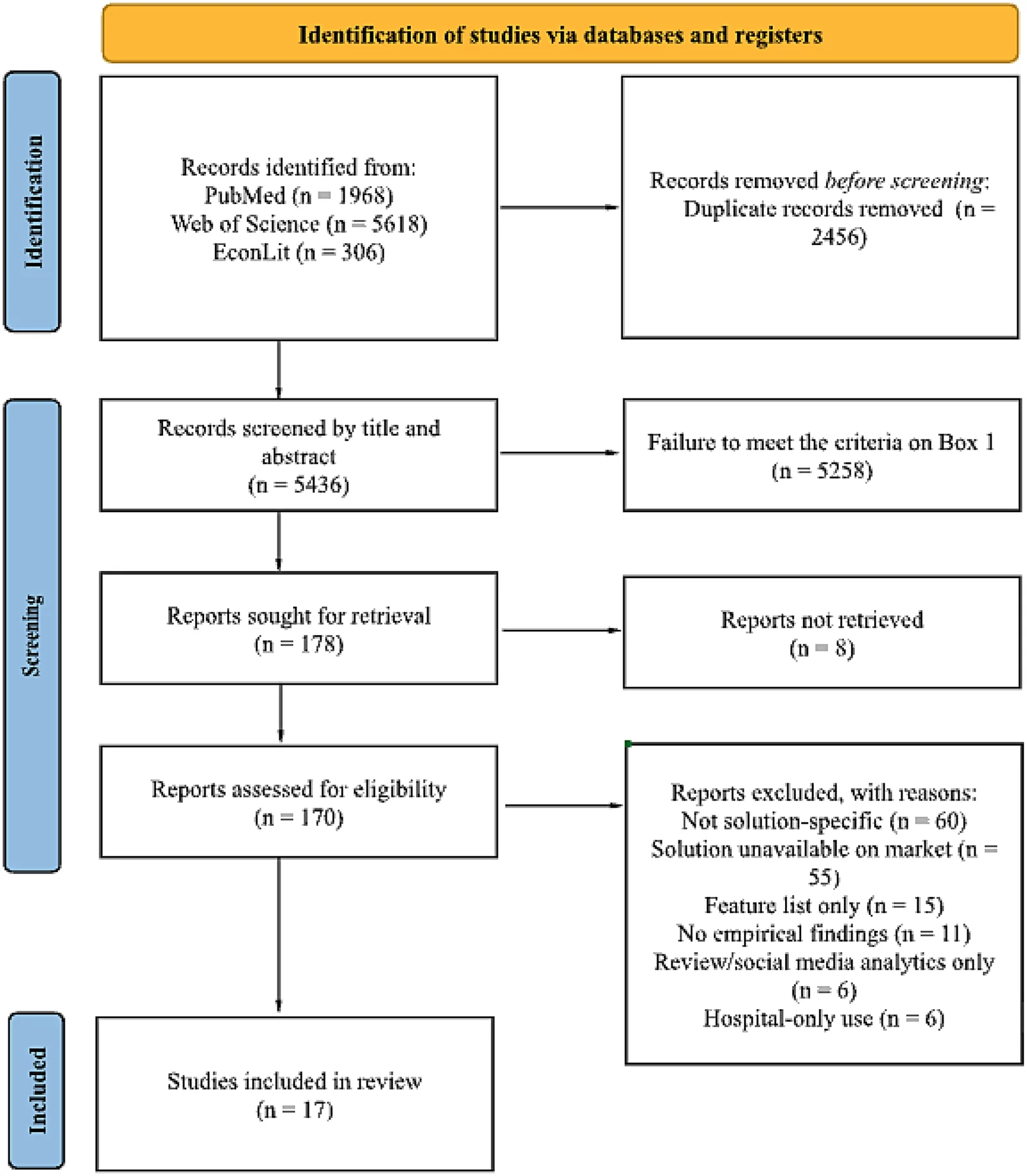
PRISMA flow diagram.

### 3.1 Included studies characterization and quality assessment

The seventeen included studies span thirteen countries, with three conducted in the United States and two in South Korea and the United Kingdom (see Supplementary File A3). Author teams were predominantly from biomedical engineering, health informatics, or health technology management (*n* = 7), psychology and behavioral sciences (*n* = 4), and clinical or public health disciplines (*n* = 4), with two studies led by teams with economics or business as the primary disciplinary background. No study was designed to examine competitive dynamics among solutions, only four explicitly addressed economic mechanisms such as willingness to pay or reimbursement. Thirteen studies analyzed solutions already available in the market; four evaluated solutions developed or deployed in academic or clinical contexts. Solutions were most often developed by private-sector agents (*n* = 12), with the remainder by public or non-profit organizations (*n* = 3) or through cross-sector collaborations (*n* = 2) (Table 2). Full study-level coding across all variables used in the synthesis is reported in Supplementary File A6.

**Table 2:** Study descriptor table.

Author, year	Value proposition	Value proposition categories	Primary agent type	Developer type	Study context	Study design
Chahal et al., 2021 (57)	Symptom management and care support for RA	Condition management	Patients	Private-sector	Market study	Quantitative descriptive
Chen et al., 2024 (58)	Cognitive support and monitoring for dementia	Condition management	Patients and caregivers	Private-sector	Market study	Quantitative descriptive
Galetsi et al., 2023 (59)	Diagnostic and educational tools for health professionals	Condition management	Health professionals	Private-sector	Market study	Quantitative descriptive
Skeens et al., 2024 (60)	Care coordination and coping support in pediatric cancer	Condition management	Patients and caregivers	Public or non-profit	Market study	Quantitative descriptive
Viswanathan et al., 2023 (61)	Geriatric assessment tools for clinical training	Condition management	Clinicians and trainees	Cross-sector	Solution evaluation	Quantitative descriptive
Richards et al., 2025 (62)	Weight management through GLP-1RA medication and digital coaching	Condition management	Patients	Private-sector	Market study	Quantitative non-randomized
Fürtjes et al., 2024 (63)	Self-guided mental well-being support	Lifestyle and behavior	General population	Private-sector	Market study	Quantitative descriptive
Hong et al., 2024 (64)	Menstrual cycle tracking and self-understanding	Lifestyle and behavior	General population	Private-sector	Market study	Mixed methods
Jakob et al., 2024 (65)	Dietary self-regulation and nutrition literacy	Lifestyle and behavior	General population	Cross-sector	Solution evaluation	Quantitative non-randomized
Kozlov et al., 2020 (66)	Mindfulness training for psychological well-being	Lifestyle and behavior	General population	Public or non-profit	Solution evaluation	Quantitative **descriptive**
Lee et al., 2020 (67)	Weight management through financial incentives	Lifestyle and behavior	General population	Private-sector	Market study	Quantitative non-randomized
Rasul et al., 2023 (68)	General health self-management	Lifestyle and behavior	General population	Private-sector	Market study	Quantitative descriptive
van der Haar et al., 2023 (69)	Nutrition self-tracking and dietary behavior change	Lifestyle and behavior	General population	Private-sector	Market study	Mixed methods
Collins, 2025 (70)	Diet quality improvement through financial incentivization	Lifestyle and behavior	General population	Public or non-profit	Market study	Quantitative non-randomized
Farič et al., 2021 (71)	Physical activity through narrative gamification	Physical activity	General population	Private-sector	Market study	Qualitative
Kanai, 2025 (72)	Physical activity through insurance-based incentives	Physical activity	General population	Private-sector	Market study	Quantitative non-randomized
Fuente-Vidal et al., 2026 (73)	Fitness app engagement and training behavior	Physical activity	General population	Private-sector	Market study	Quantitative descriptive

The seventeen studies were grouped into three value proposition categories (Table 2): condition management, encompassing solutions targeting specific clinical populations under professional or institutional oversight; lifestyle and behavior, encompassing solutions supporting self-directed personal goals without clinical integration; and physical activity, encompassing solutions whose primary value proposition is the enhancement of sports participation and physical performance. Condition management (*n* = 6) includes solutions targeting rheumatoid arthritis (RA), dementia, pediatric cancer, geriatric assessment, and GLP-1RA supported weight management. Lifestyle and behavior (*n* = 8) includes solutions addressing nutrition, weight, mental health, menstrual health, and mindfulness. Physical activity (*n* = 3) includes solutions addressing gamified physical activity, incentivized exercise engagement, and a training support app.

Study quality was assessed using MMAT (see Supplementary File A5). All seventeen studies stated clear research questions and collected appropriate data. The qualitative study met all MMAT criteria. The nine quantitative descriptive studies met criteria for sampling relevance, measurement appropriateness, and statistical analysis. Of the five quantitative non-randomized studies, representativeness was met only in one, and completeness of outcome data was met in two. The two mixed-methods studies provided adequate rationales and integration but did not fully address divergences between the qualitative and quantitative components.

### 3.2 Typified goods or services: what digital solutions are offered?

All studies examined standard mobile apps, with four exceptions: Farič and colleagues described an narrative-based narrative gamification running app (71); Collins and colleagues evaluated a website-based email nutrition challenge rather than a standalone app (70); Kanai and colleagues examined a wearable-linked insurance incentive program (72); and Richards and colleagues evaluated an app-based platform combined with prescribed medication and human health-coach support (62) (Figure 2). In condition management, each solution targeted a specific clinical population and addressed a defined functional scope, including symptom tracking, diagnostic support, care coordination, clinical training, or medication-assisted weight management. In lifestyle and behavior, solutions addressed distinct goal categories (nutrition/weight, mental health, menstrual health, and mindfulness). Across categories, however, variation in content quality and design logic was reported: most mental health apps in the lifestyle and behavior category lacked evidence of effectiveness (63), and nutrition apps showed widely varying reliability of their food databases (69), limiting cross-solution standardization. In the physical activity category, three solutions were described, spanning narrative-driven gamification, insurance-linked incentivization, and standard fitness-app training support.

**Figure 2:**
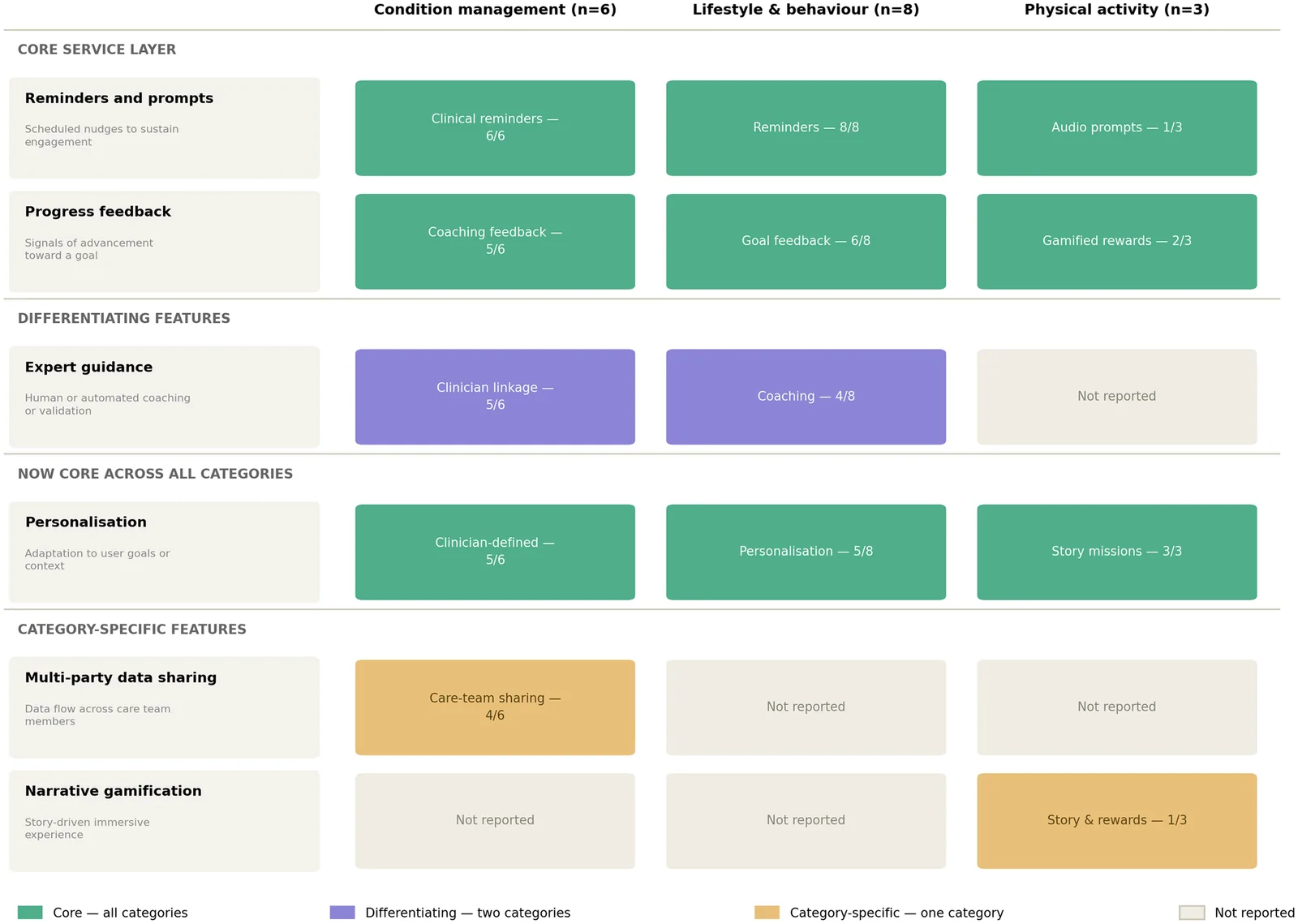
Digital solution profiles and core features by value proposition categories.

Access and pricing models differed across categories. Condition management solutions were predominantly free (*n* = 3), with one freemium, one advertising-supported, and one paid subscription model. In lifestyle and behavior, freemium was the most common model (*n* = 4), with three free solutions and two studies reporting a direct financial transfer as part of the access or incentive structure: a paid program with reimbursement contingent on adherence, and a free challenge supplemented by prize-draw incentives. The physical activity category showed mixed access models: two solutions operated on a freemium basis, with free trial or free tiers alongside paid subscription options, while the third reported a direct financial transfer through an insurance-embedded incentive structure, in which sustained engagement reduced policy premiums, rather than conventional app pricing.

### 3.3 Voluntary and specified exchange: which agents participate and under what terms?

Primary agents initiated participation voluntarily across all seventeen studies, except for three studies where clinical referral, prescription, or contractual pathways were reported: two condition management studies and one physical activity study, in which participation was embedded within an existing insurance policy relationship, partially constraining the voluntariness of the exchange (Figure 3). The condition management category described the most complete agent configuration, including, professional agents: clinicians recommending, prescribing, or validating solutions, and institutional bodies providing endorsement, co-design, or clinical oversight, reported in five of six studies. On the other hand, in lifestyle and behavior, professional and institutional agents were present in only two studies each, and in the physical activity category, professional agents were absent from all three studies, while institutional agents were reported in one, reflecting the insurance-provider role in the incentive structure. Secondary agents, caregivers supporting primary users, were reported in three condition management studies and one lifestyle study but were absent from the physical activity category.

**Figure 3:**
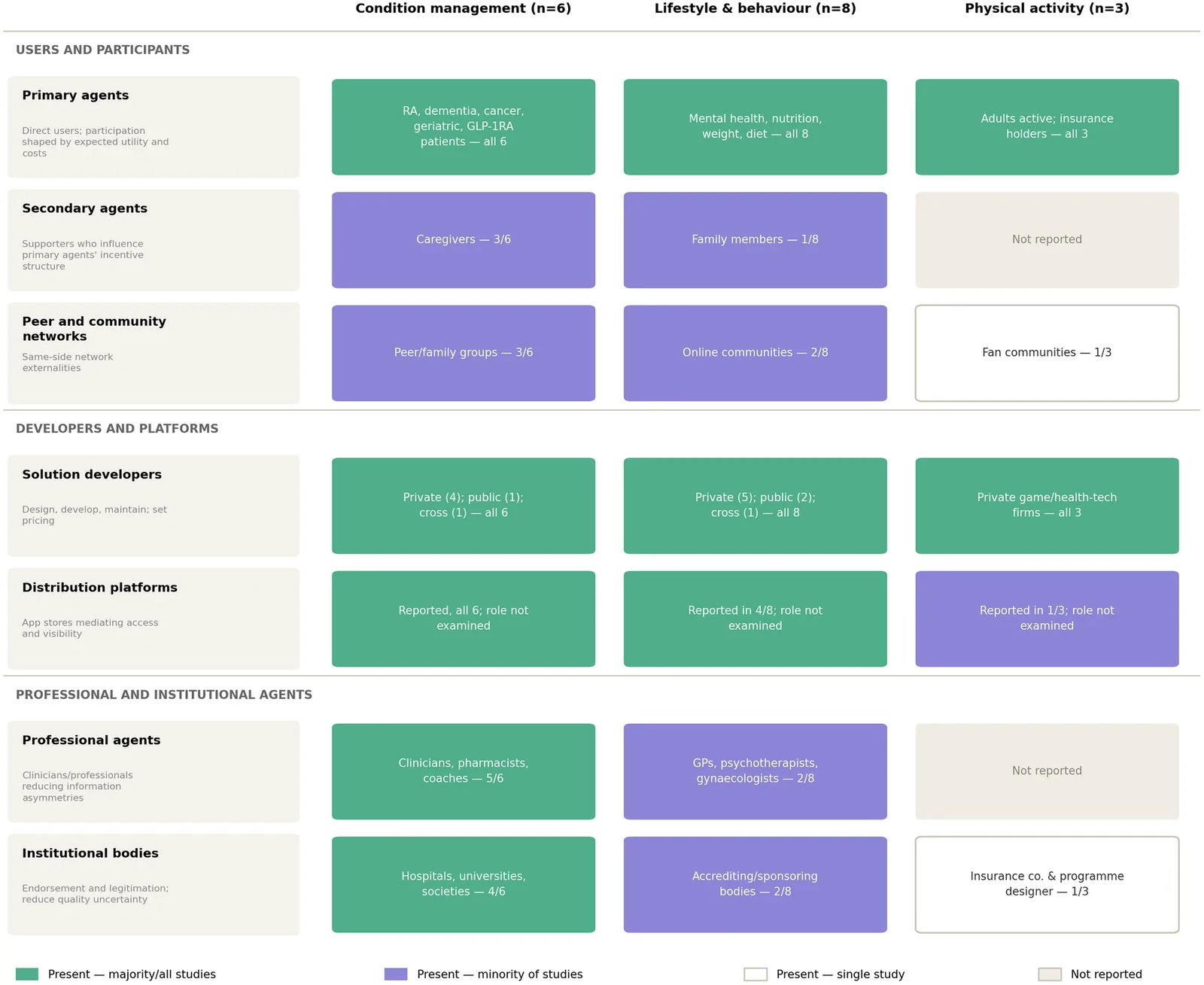
Agent types and roles by value proposition categories.

Distribution platforms mediating access and visibility were reported across all three categories, but their role was not examined in any included study. Exchange terms varied by category. In condition management, they were primarily governed by clinical protocols and care team involvement, with one study also reporting commercial subscription pricing linked to prescribed medication. In lifestyle and behavior, exchange terms were defined by app onboarding and self-selected goals, with one study incorporating incentive-linked prize draws. In physical activity, they were set by the solution's freemium structure or, in one study, by the terms of an insurance policy contract.

### 3.4 Interactions and coordination mechanisms: what types of interactions structure exchange?

Agent–solution interactions involving tracking, monitoring, reminders, and feedback were reported across all seventeen studies. Their orientation differed across categories: toward clinical care in condition management, toward personal self-management in lifestyle and behavior, and toward incentive-linked or narrative-driven experience in the physical activity category (Figure 4).

**Figure 4:**
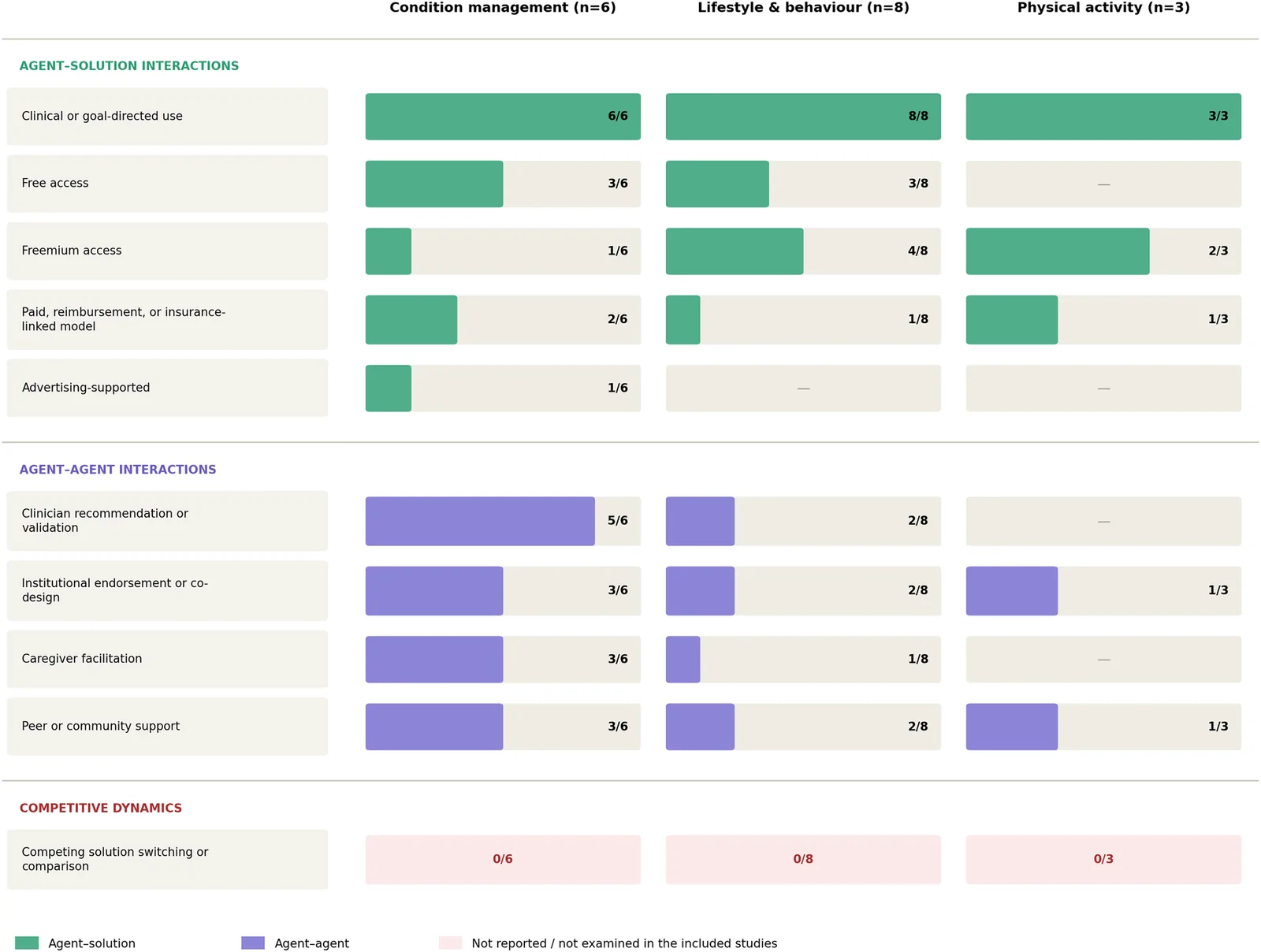
Interaction types by value proposition category.

Agent–agent interactions were most structured in condition management, where clinician recommendations, prescriptions, or validations were reported in five studies, and institutional endorsement or co-design in three. In lifestyle and behavior, professional and institutional involvement was limited to endorsement roles in two studies each, without clinical integration. In the physical activity category, agent–agent interactions were reported in one of three studies, limited to peer encouragement, with no agent–agent ties reported in the remaining two.

### 3.5 Regularity and competition: what factors shape agents’ participation over time?

Participation drivers were reported only from the primary agent side across all seventeen studies (Figure 5). Perceived value and usefulness were the most consistently reported entry drivers across the condition management and lifestyle and behavior categories, indicating that expected benefit is the primary signal shaping adoption decisions regardless of context. Institutional endorsement was a prominent entry driver in condition management (*n* = 4) but was reported in two lifestyle studies and one physical activity study, reflecting the differential role of professional legitimation across categories.

**Figure 5:**
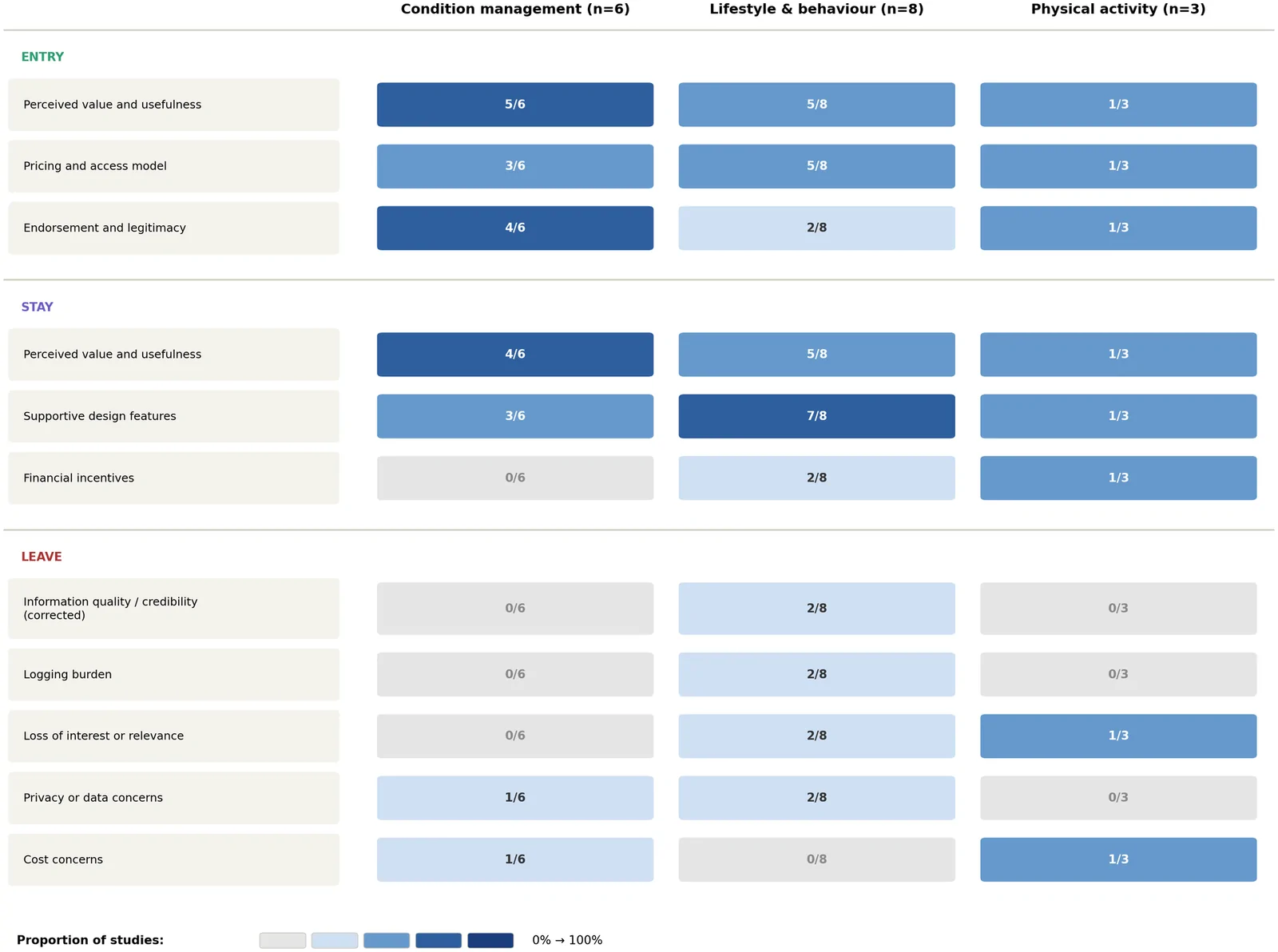
Participation drivers across entry, retention, and exit stages by value proposition category.

Supportive design features, reminders, feedback, and coaching were reported as a driver of continued use in two lifestyle and behavior studies, with no condition management study reporting this driver, making them the most consistently coded retention mechanism in the evidence base within that category. Financial mechanisms explicitly sustaining participation were reported in three studies, spanning two categories: Lee et al. (2020) and Collins et al. (2025) in lifestyle and behavior, where monetary reimbursement and prize-draw incentives respectively were associated with continued engagement. Discontinuation drivers differed across categories. In lifestyle and behavior, exit was associated with information quality, credibility, or content quality concerns (*n* = 2), including inconsistent calorie/nutrient values in the app's food-logging database and a lack of new information over time, logging burden (*n* = 2), and privacy or data concerns (*n* = 1). In physical activity, exit drivers included cost concerns and loss of interest. Condition management studies did not report exit drivers, with one exception: Richards et al. (2025) reported detailed withdrawal reasons among the 60.4% of participants who became inactive at 12 months, the most commonly cited being cost (*n* = 82) and side effects (*n* = 54), followed by personal reasons (*n* = 22), lack of progress (*n* = 17), goal achievement (*n* = 15), service issues (*n* = 5), and other or unspecified reasons (*n* = 11). No study across any category reported discontinuation driven by agents switching to a competing solution or responding to the pricing of alternatives.

### 3.6 Marketness assessment

Voluntary exchange is present across most of lifestyle and behavior and physical activity, where participation is typically freely initiated and exchange terms are specified through commercial access models; the exception is Kanai et al. (2025), where participation was embedded within an existing insurance policy relationship, partially constraining voluntariness within the physical activity category. Condition management sits closer to the hierarchy end: clinical referral or prescription pathways constrained voluntary participation in two studies, and exchange terms were governed by clinical protocols rather than freely negotiated in most of the category, though one study (Richards et al., 2025) involved a freely negotiated commercial subscription.

Regarding typified goods or services, all three categories sit in an intermediate position, though for different reasons. Condition management is limited by heterogeneity across clinical conditions, now spanning both clinician-integrated tools and a pharmacologically supported weight-management service. Lifestyle and behavior is limited by variation in content quality, design logic, and delivery format, spanning both app-based and website-based solutions. Physical activity spans three structurally distinct types: a narrative-driven app, an insurance-embedded incentive program, and a conventional fitness-tracking app, making it the least typified of the three categories.

Regularity follows a related but more varied pattern than a single mechanism per category. In condition management, it is predominantly institutionally dependent, with disruption reported through cost and treatment-related concerns; credibility concerns were not reported in this category and instead apply to lifestyle and behavior, where regularity is largely design-dependent, with one study additionally sustained through financial incentives, and disrupted by logging burden, information-quality concerns, and motivational decay. In physical activity, regularity mechanisms diverge by solution type, design-dependent in one study, financial-incentive-dependent in another, and motivation-dependent in the third, with disruption documented only for the original narrative-driven solution (cost concerns and loss of interest).

Competition is absent from the evidence base across all three categories. No included study examined agent switching, price pressure, or any competitive dynamics. This reflects a research gap rather than an absence of competitive dynamics: solution proliferation is documented across all three categories, suggesting competing alternatives exist but have not been studied as such.

Overall, condition management is best characterized as a hybrid form approaching the hierarchy end. Lifestyle and behavior is best characterized as a hybrid form approaching the market end. Physical activity spans a wider range of market-like characteristics across its three constituent studies, from a freely negotiated freemium model to an institutionally embedded incentive structure and is best characterized as a heterogeneous hybrid form rather than positioned at a single point on the continuum (Figure 6).

**Figure 6:**
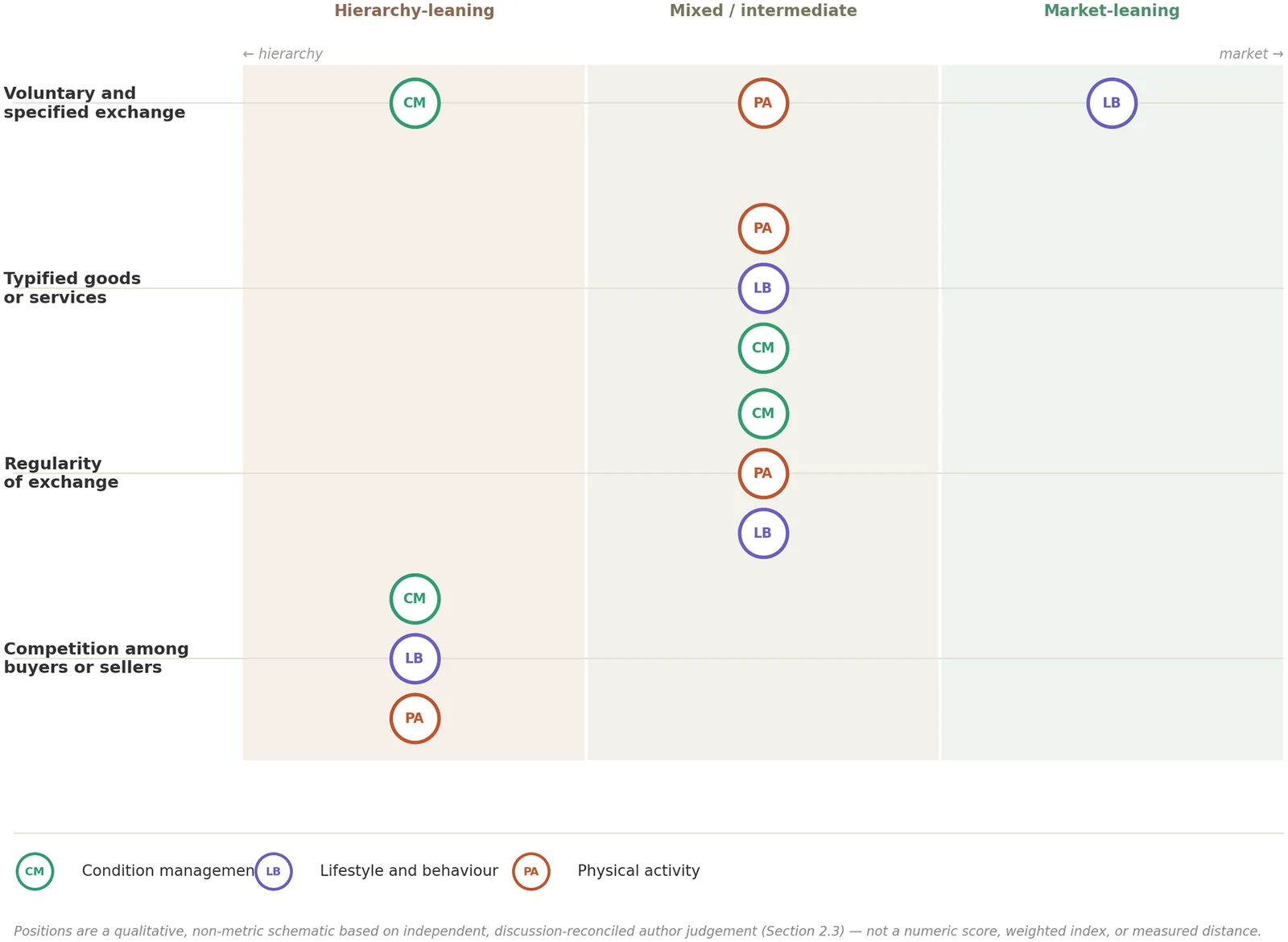
Positioning of value proposition categories along the marketness continuum.

## 4 Discussion

This systematic review applied Rosenbaum's marketness framework to digital health, sports, and well-being settings for the first time. No value proposition category met all four criteria for a fully constituted market. Condition management was positioned closer to the hierarchy end: clinical governance constrains voluntary exchange, and regularity depends on institutional endorsement rather than price signals. Lifestyle and behavior was positioned closer to the market end: exchange terms are commercial, and participation is freely initiated, but logging burdens, motivational decay, and privacy concerns disrupt regularity. Across all three categories, the competition criterion was absent from the evidence base. No included study examined agent switching, price pressure among developers, or any competitive dynamic. These findings suggest a reframing of the sustained participation gap described in the introduction. Rather than attributing this gap solely to poor app design, our findings point to a broader empirical gap: the economic mechanisms that could, in principle, coordinate sustained participation, competition, pricing, and supply-side responses have rarely been examined in this literature. This review cannot establish that these mechanisms causally explain observed discontinuation, only that their absence from the evidence base leaves the question open.

Condition management's apparent reliance on institutional endorsement rather than price signals is consistent with existing work on experience goods and credence goods in health markets (74), where quality uncertainty creates demand for trusted third-party signals. Digital health users cannot assess whether an app is clinically sound before downloading it, and unlike traditional healthcare, there is no prescribing physician or regulatory stamp by default. The digital health literature has documented reliance on professional society endorsements and clinical recommendations (50). Our marketness assessment suggests that this reliance may reflect more than a scattered design choice or a sign of market immaturity: it is consistent with a structural response to information asymmetry, and it is this pattern that positions condition management closer to the hierarchy end of the continuum. The included studies were not designed to test this explanation directly, however, and alternative accounts (e.g., regulatory convention, clinical liability norms) cannot be ruled out. This aligns with Vaissière and Levrel's (2015) assessment of biodiversity offset markets, where weak competition and irregular exchange forced coordination through institutional authority rather than prices (28). In both contexts, partial marketness suggests that analytical approaches focused on signaling, institutional coordination, and principal-agent relationships may be more informative than approaches relying primarily on price-based coordination.

In the context of lifestyle and behavior, research on digital goods has established that free tiers serve as trial mechanisms that generate demand-side data, but users rarely understand the value of their own data as a form of payment (75). Our results show that this data-as-currency ambiguity may weaken the voluntariness criterion of marketness, a market failure previously documented in social media and online platform contexts but not in this one (76). In addition, the logging burdens and motivational decay we identified echo behavioral economics research on attrition in commitment devices (77). Unlike clinical settings where professional oversight provides external accountability, lifestyle apps rely entirely on user self-regulation, which is systematically biased toward dropout. This is consistent with the possibility that the regularity criterion of marketness reflects behavioral as well as design factors, potentially related to present bias and time-inconsistent preferences (78), although the included studies did not directly measure these constructs (74). This present-bias account, however, may itself conflate two distinct phenomena: a committed user losing motivation during use, and a user who had not yet formed a stable intention to adopt the behavior in the first place, a distinction tied to readiness to change that this review's evidence cannot resolve. Content quality concerns, reported alongside these behavioral drivers, indicate that regularity in this category is also disrupted by the substance of what is delivered, not solely by usability or user self-regulation. Finally, indirect evidence of platform interdependencies, caregivers and clinicians influencing participation, aligns with platform economics literature, but no existing study has measured these cross-side effects.

Taken together, the categories reveal a broad contrast in their coordination mechanisms: where institutional authority is strong, market mechanisms are weak, and vice versa. Condition management appears to rely on clinical endorsement in place of price-based coordination, consistent with the expectation that quality is difficult to observe without professional mediation (70); this review did not test whether pricing mechanisms could, in principle, achieve similar coordination. Lifestyle and behavior operates with commercial pricing but continues to report factors, present bias, logging fatigue, and privacy opacity that may disrupt regular participation. Physical activity does not fit either pattern cleanly: its three constituent studies span a freely negotiated freemium model, an institutionally embedded insurance incentive, and a conventional fitness-tracking app, suggesting that coordination mechanisms in this category may be solution-specific rather than category-wide; given the small number of studies, this heterogeneity should be treated as a preliminary observation rather than an established pattern. This marketness positioning also has implications for the types of regulatory instruments that may warrant consideration. Condition management, near the hierarchy end, instruments commonly used for credence goods, such as quality certification, clinical validation, or prescribing guidance, may be more relevant than approaches that rely primarily on competitive price signals. Price-based regulation would be premature without evidence of competition. Lifestyle and behavior, closer to the market end, presents a different challenge: data-as-currency ambiguity suggests that mechanisms supporting transparency and informed consent may be particularly relevant. Physical activity's heterogeneity suggests that a uniform regulatory approach may be premature for this category specifically, pending evidence on whether its solution types converge toward a common coordination mechanism as the evidence base grows. More broadly, applying the same regulatory tools across all categories risks assuming market conditions that the available evidence does not establish.

Several limitations bound this review. It does not claim that competition is absent from these markets, only that no included study examined it. Whether competitive dynamics exist remains an open empirical question. This may partly reflect our search strategy, which did not explicitly include competition-related concepts. Nevertheless, the eligibility criteria and inductive coding framework were designed to capture competitive dynamics, such as agent switching or price pressure, wherever reported. The absence of these dynamics across all seventeen studies is consistent with solution proliferation being widely documented, while digital health, sports, and well-being solutions have rarely been studied as competing market alternatives. The physical activity category spans three structurally distinct studies; findings are reported descriptively and should be interpreted with caution when generalizing to digital sports markets. The small size of this category may partly reflect our search strategy's disciplinary coverage. Nevertheless, the same eligibility criteria and screening procedure were applied uniformly across all three value proposition categories, and no sport-specific search terms were required to identify the studies ultimately included in this category. More broadly, the category-level evidence base remains small: condition management (*n* = 6), lifestyle and behavior (*n* = 8), and physical activity (*n* = 3) are each characterized by a limited number of studies, such that the addition or exclusion of a single study could plausibly shift a category's positioning along the marketness continuum. This positioning should therefore be read as indicative of current evidence rather than a stable or precise placement. The search was restricted to English-language articles published between 2020 and June 2026. EconLit was included but returned few relevant studies. This review's marketness assessment also does not incorporate heterogeneity in individual readiness to change. Marketness characterizes the setting in which exchange occurs, not the psychological state of individual participants, and it is unclear whether a framework built around the structure of exchange can accommodate this kind of individual-level variation, or whether it lies outside what marketness, as an analytical tool, is designed to capture. Within these boundaries, the central finding stands: the economic mechanisms that sustain participation remain empirically uncharacterized.

These limitations point to four priorities for future research: testing whether participation reflects comparative choice among alternatives, estimating the implicit price users place on their data, identifying whether developers respond to competition at all, and testing whether accountability mechanisms improve retention where present bias drives attrition. Testing whether participation reflects comparative choice among alternatives follows from condition management's apparent reliance on institutional endorsement, which predicts that users treat endorsed solutions as substitutes for each other, not for non-endorsed apps; this could be tested with discrete-choice experiments or revealed-preference switching data, though such data may itself be scarce, a null result that would support the account, while evidence of switching among non-endorsed apps would suggest institutional endorsement is not, in fact, structurally necessary. Estimating the implicit price users place on their data follows from the data-as-currency ambiguity identified in lifestyle and behavior; conjoint experiments contrasting subscription and data-funded tiers could recover this willingness to pay, though incentive-compatible designs would be needed given the risk of hypothetical bias, and a substantial gap between users’ implicit valuation and the data's market value to developers would provide direct evidence of the voluntariness violation this review only infers indirectly. Identifying whether developers respond to competition at all follows directly from the absence of any evidence on competition in the included studies; longitudinal panel data on pricing and entry within a category could test this, though such data is typically proprietary and may require direct developer partnerships to obtain, and evidence of price or feature responses to entrants would indicate a degree of market function that our synthesis, limited to user-side evidence, could not establish. Finally, the present-bias account of attrition motivates the final priority above: comparing retention between lifestyle and behavior solutions that do and do not include third-party accountability features, such as caregiver or clinician check-ins, an approach constrained by how few existing platforms vary this feature in a way that permits clean comparison; a retention gap favoring accountability features would suggest the regularity criterion is shaped by institutional design choices, not only by individual behavioral traits. Any such comparison should also account for heterogeneity in readiness to change, since an accountability mechanism that improves retention among users already committed to sustained use may have little or no effect on users who have not yet formed the intention to adopt the behavior in the first place, and pooling these groups could mask the mechanism's true effect.

Overall, this review aimed to assess the extent to which digital health, sports, and well-being solutions display the characteristics of a market across condition management, lifestyle and behavior, and physical activity, and found that none met all four marketness criteria, with competition remaining an evidence gap rather than a demonstrated absence across all three. Sustained participation in these solutions is often attributed to design, usability, or content failures alone, but these findings point instead to a broader empirical gap: the economic mechanisms that could, in principle, sustain participation have rarely been studied as such. Addressing this gap requires testing whether participation reflects genuine comparative choice, estimating the price users implicitly place on their data, establishing whether developers respond to competition, and testing whether accountability mechanisms can substitute for institutional endorsement. Such evidence is needed before investment, regulatory, and design decisions can be grounded in an empirically characterized understanding of market function.

## Data Availability

The raw data supporting the conclusions of this article will be made available by the authors, without undue reservation.
