# Supplementary material for: Beyond the app: a marketness assessment of digital health, sports, and well-being - a systematic review
Source: Front Digit Health. 2026 Oct 8;8:1871114. doi: 10.3389/fdgth.2026.1871114 (PMC13687406; doi:10.3389/fdgth.2026.1871114)
Supplement: Supplementary file 1 [file Datasheet1.pdf]

Supplementary Material A1: PRISMA checklist.

Section and Topic	Item #	Checklist Item	Location in Manuscript
**TITLE**			
Title	**1**	Identify the report as a systematic review.	Title page (p. 1)
**ABSTRACT**			
Abstract	**2**	See the PRISMA 2020 for Abstracts checklist.	Abstract (p. 1)
**INTRODUCTION**			
Rationale	**3**	Describe the rationale for the review in the context of existing knowledge.	Introduction (pp. 2–3)
Objectives	**4**	Provide an explicit statement of the objective(s) or question(s) the review addresses.	Introduction, Table 1 (p. 4)
**METHODS**			
Eligibility criteria	**5**	Specify the inclusion and exclusion criteria for the review and how studies were grouped for the syntheses.	Section 2.1.2, Box 1 (p. 5)
Information sources	**6**	Specify all databases, registers, websites, organisations, reference lists and other sources searched or consulted. Specify the date when each source was last searched.	Section 2.1.1 (p. 4)
Search strategy	**7**	Present the full search strategies for all databases, registers and websites, including any filters and limits used.	Section 2.1.1 (p. 4); full strings in Supplementary Material A2
Selection process	**8**	Specify the methods used to decide whether a study met the inclusion criteria, including how many reviewers screened each record, whether they worked independently, and details of automation tools used.	Section 2.1.2 (p. 5)
Data collection process	**9**	Specify the methods used to collect data from reports, including how many reviewers collected data, whether they worked independently, and any processes for obtaining or confirming data from study investigators.	Section 2.2 (p. 6)
Data items - outcomes	**10a**	List and define all outcomes for which data were sought.	Sections 2.2.1–2.2.4 (pp. 6–7)
Data items - other variables	**10b**	List and define all other variables for which data were sought (e.g. participant and intervention characteristics, funding sources).	Section 2.2 (p. 6)
Study risk of bias assessment	**11**	Specify the methods used to assess risk of bias in the included studies, including details of the tool(s) used, how many reviewers assessed each study and whether they worked independently.	Section 2.1.3 (p. 6)
Effect measures	**12**	Specify for each outcome the effect measure(s) used in the synthesis or presentation of results.	Not applicable: qualitative synthesis
Synthesis methods - eligibility	**13a**	Describe the processes used to decide which studies were eligible for each synthesis.	Section 2.3 (p. 7)

Synthesis methods - data preparation	**13b**	Describe any methods required to prepare the data for presentation or synthesis.	Section 2.2 (p. 6)
Synthesis methods - display	**13c**	Describe any methods used to tabulate or visually display results of individual studies and syntheses.	Section 2.3 (p. 7)
Synthesis methods - rationale	**13d**	Describe any methods used to synthesize results and provide a rationale for the choice(s). If meta-analysis was performed, describe the model(s) and method(s).	Section 2.3 (p. 7)
Synthesis methods - heterogeneity	**13e**	Describe any methods used to explore possible causes of heterogeneity among study results.	Addressed narratively, Section 2.3 (p. 7)
Synthesis methods - sensitivity	**13f**	Describe any sensitivity analyses conducted to assess robustness of the synthesized results.	Not conducted. The qualitative and narrative nature of the synthesis did not lend itself to sensitivity analyses.
Reporting bias assessment	**14**	Describe any methods used to assess risk of bias due to missing results in a synthesis (arising from reporting biases).	Not explicitly addressed. The discussion notes that EconLit returned few relevant studies, suggesting potential publication bias against economic analyses of digital health settings. Authors should consider addressing this more directly.
Certainty assessment	**15**	Describe any methods used to assess certainty (or confidence) in the body of evidence for an outcome.	Section 2.1.3 (p. 5): MMAT used for quality appraisal. No formal certainty framework (e.g., GRADE) applied, consistent with the qualitative synthesis approach. MMAT results reported narratively in Section 3.1 and Supplementary Material A5.
**RESULTS**			
Study selection	**16a**	Describe the results of the search and selection process, from the number of records identified in the search to the number of studies included in the review, ideally using a flow diagram.	Section 3, Figure 1 (p. 8)
Study selection - excluded	**16b**	Cite studies that might appear to meet the inclusion criteria, but which were excluded, and explain why they were excluded.	Section 3, Figure 1 (p. 8)
Study characteristics	**17**	Cite each included study and present its characteristics.	Section 3.1, Table 2 (pp. 9–10)
Risk of bias in studies	**18**	Present assessments of risk of bias for each included study.	Section 3.1 (p. 9); full assessments in Supplementary Material A5
Results of individual studies	**19**	For all outcomes, present, for each study: (a) summary statistics for each group and (b) an effect estimate and its precision.	Not applicable: narrative synthesis; individual contributions in Sections 3.2–3.5 (pp. 12–16) and Supplementary Material A6

Results of syntheses	**20a**	Briefly summarise the characteristics and risk of bias among contributing studies.	Section 3.1 (p. 9)
Results of syntheses - statistical	**20b**	Present results of all statistical syntheses conducted.	Not applicable. No statistical synthesis conducted.
Results of syntheses - heterogeneity	**20c**	Present results of all investigations of possible causes of heterogeneity among study results.	Not applicable. Addressed narratively through cluster-level analysis.
Results of syntheses - sensitivity	**20d**	Present results of all sensitivity analyses conducted.	Not applicable. No sensitivity analyses conducted.
Reporting biases	**21**	Present assessments of risk of bias due to missing results (arising from reporting biases) for each synthesis assessed.	Not explicitly presented. The discussion acknowledges EconLit returned few results and that no study examined competitive dynamics, which may reflect publication or disciplinary bias. Authors should consider a more explicit assessment.
Certainty of evidence	**22**	Present assessments of certainty (or confidence) in the body of evidence for each outcome assessed.	Not formally assessed using a certainty framework. The marketness continuum positioning in Section 3.6 and Figure 6 provides an implicit characterisation of evidence strength, distinguishing between genuine absence of market features and absence of research on those features.
**DISCUSSION**			
Discussion - interpretation	**23a**	Provide a general interpretation of the results in the context of other evidence.	Section 4 (pp. 19–21)
Discussion - evidence limitations	**23b**	Discuss any limitations of the evidence included in the review.	Discussion, Limitations (p. 20–21)
Discussion - review limitations	**23c**	Discuss any limitations of the review processes used.	Discussion, Limitations (p. 20–21); Section 2.1 (p. 4) for non-registration
Discussion - implications	**23d**	Discuss implications of the results for practice, policy, and future research.	Section 4 (pp. 21–22), future research priorities
**OTHER INFORMATION**			
Registration - registration	**24a**	Provide registration information for the review, including register name and registration number, or state that the review was not registered.	Section 2.1 (p. 4)
Registration - protocol	**24b**	Indicate where the review protocol can be accessed, or state that a protocol was not prepared.	Section 2.1 (p. 4)
Registration - amendments	**24c**	Describe and explain any amendments to information provided at registration or in the protocol.	Not applicable
Support	**25**	Describe sources of financial or non-financial support for the review, and the role of the funders or sponsors in the review.	Section 7, Funding (p. 22)
Competing interests	**26**	Declare any competing interests of review authors.	Section 5, Conflict of Interest (p. 22)

Data availability	**27**	Report which of the following are publicly available and where they can be found: template data collection forms; data extracted from included studies; data used for all analyses; analytic code; any other materials used.	Section 11, Data Availability Statement (p. 28)
