# Supplementary material for: Beyond the app: a marketness assessment of digital health, sports, and well-being - a systematic review
Source: Front Digit Health. 2026 Oct 8;8:1871114. doi: 10.3389/fdgth.2026.1871114 (PMC13687406; doi:10.3389/fdgth.2026.1871114)
Supplement: Supplementary file 2 [file Datasheet2.pdf]

Supplementary Material A2: Queries used to search each database.

Pubmed

#1	("Buyer"[MeSH Terms] OR "Seller"[Title/Abstract] OR "Consumer"[Title/Abstract] OR "Producer"[Title/Abstract] OR "Stakeholder"[Title/Abstract] OR "Social Interaction"[MeSH Terms] OR "Coordination"[Title/Abstract] OR "Cooperation"[Title/Abstract] OR "Social Structure"[MeSH Terms] OR "Peer Influence"[MeSH Terms] OR "Digital market"[Title/Abstract] OR "Platform market"[Title/Abstract] OR "Economic action"[Title/Abstract] OR "Economic behavior"[Title/Abstract] OR "Economic behaviour"[Title/Abstract] OR "Platform economy"[Title/Abstract] OR "Willingness to pay"[Title/Abstract] OR "Negotiating"[MeSH Terms] OR "Commerce"[MeSH Terms] OR "Resource Allocation"[MeSH Terms] OR "Incentives"[Title/Abstract] OR "Gamification"[MeSH Terms] OR "downloads"[Title/Abstract] OR "user base"[Title/Abstract] OR "active users"[Title/Abstract] OR "audience size"[Title/Abstract] OR "app usage"[Title/Abstract] OR "feature changes"[Title/Abstract] OR "app updates"[Title/Abstract] OR "version history"[Title/Abstract] OR "product evolution"[Title/Abstract] OR "pricing model"[Title/Abstract] OR "subscription"[Title/Abstract] OR "freemium"[Title/Abstract] OR "in-app purchase"[Title/Abstract] OR "monetization"[Title/Abstract] OR "revenue model"[Title/Abstract] OR "pricing strategy"[Title/Abstract])
#2	("Cybersport"[Title/Abstract] OR "Cyber sport"[Title/Abstract] OR "Virtual sport"[Title/Abstract] OR "Digital sport"[Title/Abstract] OR "Exergaming"[MeSH Terms] OR "Active video game"[Title/Abstract] OR "Active video games"[Title/Abstract] OR ("Sport"[Title/Abstract] AND "digit*"[Title/Abstract]) OR "Digital Health"[MeSH Terms] OR "Digital health technology"[Title/Abstract] OR "Digital medical device"[Title/Abstract] OR "Digital medical devices"[Title/Abstract] OR "Digital care"[Title/Abstract] OR "eHealth"[Title/Abstract] OR "e-health"[Title/Abstract] OR "Mobile health"[Title/Abstract] OR "mHealth"[Title/Abstract] OR "m-health"[Title/Abstract] OR "Virtual health"[Title/Abstract])
#3	("Mental Health"[MeSH Terms] OR "Wellbeing"[Title/Abstract] OR "well being"[Title/Abstract] OR "Well-being"[Title/Abstract] OR "Psychological Well-Being"[MeSH Terms] OR "Personal Satisfaction"[Title/Abstract] OR "Coping Skills"[MeSH Terms] OR "Work-Life Balance"[MeSH Terms] OR "Quality of Life"[MeSH Terms] OR "Emotional awareness"[Title/Abstract] OR "Emotional fitness"[Title/Abstract] OR "Emotional Intelligence"[MeSH Terms] OR "Emotional Regulation"[MeSH Terms] OR "Emotional control"[Title/Abstract] OR "Emotional management"[Title/Abstract] OR "Emotional stability"[Title/Abstract] OR "Emotional security"[Title/Abstract] OR "Emotional Adjustment"[MeSH Terms] OR "Emotional function"[Title/Abstract] OR "Selfcare"[Title/Abstract] OR "Self care"[MeSH Terms] OR "Wellness"[Title/Abstract] OR "well-ness"[Title/Abstract])
#4	("smartphone app*"[Title/Abstract] OR "mobile app*"[MeSH Terms] OR "mobile phone app*"[Title/Abstract] OR "mobile device app*"[Title/Abstract] OR "portable software app*"[Title/Abstract] OR "tablet app*"[Title/Abstract] OR "digital intervention"[Title/Abstract] OR "digital treatment"[Title/Abstract] OR "digital

	app*"[Title/Abstract] OR "digital mental health intervention"[Title/Abstract])
	1. #3 AND #4 -> #5<br>2. #2 OR #5 -> #6<br>3. #1 AND #6 -> #7
	Filters:<br>- 2020-Juno 2026<br>- English<br>- Humans

Web of Science:

#1	TS=("Buyer" OR "Seller" OR "Consumer" OR "Producer" OR "Stakeholder" OR "Social Interaction" OR "Coordination" OR "Cooperation" OR "Social Structure" OR "Peer Influence" OR "Digital market" OR "Platform market" OR "Economic action" OR "Economic behavior" OR "Economic behaviour" OR "Platform economy" OR "Willingness to pay" OR "Negotiating" OR "Commerce" OR "Resource Allocation" OR "Incentives" OR "Gamification" OR "downloads" OR "user base" OR "active users" OR "audience size" OR "app usage" OR "feature changes" OR "app updates" OR "version history" OR "product evolution" OR "pricing model" OR "subscription" OR "freemium" OR "in-app purchase" OR "monetization" OR "revenue model" OR "pricing strategy")
#2	TS=("Cybersport" OR "Cyber sport" OR "Virtual sport" OR "Digital sport" OR "Exergaming" OR "Active video game" OR "Active video games" OR ("Sport" AND "digit*") OR "Digital Health" OR "Digital health technology" OR "Digital medical device" OR "Digital medical devices" OR "Digital care" OR "eHealth" OR "e-health" OR "Mobile health" OR "mHealth" OR "m-health" OR "Virtual health")
#3	TS=("mental health" OR "Wellbeing" OR "well being" OR "Well-being" OR "Psychological Well-Being" OR "Personal Satisfaction" OR "Coping Skills" OR "Work-Life Balance" OR "Quality of Life" OR "Emotional awareness" OR "Emotional fitness" OR "Emotional Intelligence" OR "Emotional Regulation" OR "Emotional Control" OR "Emotional management" OR "Emotional stability" OR "Emotional security" OR "Emotional Adjustment" OR "Emotional Function" OR "Selfcare" OR "Self care" OR "Self-care" OR "Wellness" OR "well-ness")
#4	TS=("smartphone app*" OR "mobile app*" OR "mobile phone app*" OR "mobile device app*" OR "portable software app*" OR "tablet app*" OR "digital intervention" OR "digital treatment" OR "digital app*" OR "digital mental health intervention")
	4. #3 AND #4 -> #5<br>5. #2 OR #5 -> #6<br>6. #1 AND #6 -> #7
	Filters:<br>- 2020-Juno 2026<br>- English

Econlit:

#1	TX ("buyer" OR seller OR consumer OR producer OR stakeholder OR "social interaction" OR coordination OR cooperation OR "social structure" OR "peer influence" OR "digital market" OR "platform market" OR "economic action" OR "economic behavior" OR "economic behaviour" OR "platform economy" OR "willingness to pay" OR negotiating OR commerce OR "resource allocation" OR incentives OR gamification OR downloads OR "user base" OR "active users" OR "audience size" OR "app usage" OR "feature changes" OR "app updates" OR "version history" OR "product evolution" OR "pricing model" OR subscription OR freemium OR "in-app purchase*" OR monetization OR "revenue model" OR "pricing strateg*")
#2	TX (cybersport OR "cyber sport" OR "virtual sport*" OR "digital sport*" OR exergam* OR "active video game*" OR (sport* N3 digit*) OR "digital health" OR "digital health technolog*" OR "digital medical device*" OR "digital care" OR eHealth OR e-health OR "mobile health" OR mHealth OR m-health OR "virtual health")
#3	TX ("mental health" OR wellbeing OR "well being" OR "well-being" OR "psychological well-being" OR "personal satisfaction" OR "coping skills" OR "work-life balance" OR "quality of life" OR "emotional awareness" OR "emotional fitness" OR "emotional intelligence" OR "emotional regulation" OR "emotional control" OR "emotional management" OR "emotional stability" OR "emotional security" OR "emotional adjustment" OR "emotional function" OR selfcare OR "self care" OR wellness OR "well-ness")
#4	TX ("smartphone app*" OR "mobile app*" OR "mobile phone app*" OR "mobile device app*" OR "portable software app*" OR "tablet app*" OR "digital intervention*" OR "digital treatment*" OR "digital app*" OR "digital mental health intervention*")
	7. #3 AND #4 -> #5<br>8. #2 OR #5 -> #6<br>9. #1 AND #6 -> #7
	Filters:<br>- 2020-Juno 2026<br>- English
