# Supplementary material for: Beyond the app: a marketness assessment of digital health, sports, and well-being - a systematic review
Source: Front Digit Health. 2026 Oct 8;8:1871114. doi: 10.3389/fdgth.2026.1871114 (PMC13687406; doi:10.3389/fdgth.2026.1871114)
Supplement: Supplementary file 3 [file Datasheet3.pdf]

Supplementary Material A3: Author and Affiliation Characteristics of Included Studies

Chahal et al., 2021

Authors listed: 3

#	Author Name	Gender	Nationality	Affiliation	Affiliation Theme
1st	Simran Chahal	Female	United Kingdom	Augurex Life Sciences Corp., Vancouver, British Columbia, Canada	Biomedical Engineering & Medical Devices
2nd	Norma Biln	Female	Canada	Augurex Life Sciences Corp., Vancouver, British Columbia, Canada	Biomedical Engineering & Medical Devices
Last	Bruce Clarke	Male	Canada	B2 Consulting, Kelowna, British Columbia, Canada	Economics & Business

Chen et al., 2024

Authors listed: 3

#	Author Name	Gender	Nationality	Affiliation	Affiliation Theme
1st	Tzu Han Chen	Male	China	PhD Program for Health Science and Industry, China Medical University, Taichung, Taiwan	Health & Technology Management
2nd	Shin-Da Lee	Male	China	PhD Program in Healthcare Science, Department of Physical Therapy, China Medical University, Taichung, Taiwan	Biomedical Engineering & Medical Devices
Last	Wei-Fen Ma	Female	China	PhD Program in Healthcare Science, School of Nursing, China Medical University, Taichung, Taiwan	Nursing & Health Care Sciences

Farič et al., 2021

Authors listed: 6

#	Author Name	Gender	Nationality	Affiliation	Affiliation Theme
1st	Nusˇa Faricˇ	Female	United Kingdom	Department of Behavioural Science and Health, University College London, London, United Kingdom.	Clinical Psychology & Behavioral Science
2nd	Henry W.W. Potts	Male	United Kingdom	Institute of Health Informatics, University College London, United Kingdom.	Digital Health & Health Informatics
3rd	Sarah Rowe	Female	New Zealand	Division of Psychiatry, University College London, United Kingdom.	Clinical Psychology & Behavioral Science
4th	Taryn Beaty	Female	United Kingdom	Six to Start, LSD Accountants, London, United Kingdom.	Economics & Business
5th	Adrian Hon	Male	United Kingdom	Six to Start, LSD Accountants, London, United Kingdom.	Economics & Business
Last	Abi Fisher	Female	United Kingdom	Department of Behavioural Science and Health, University College London, London, United Kingdom.	Clinical Psychology & Behavioral Science

Fürtjes et al., 2024

Authors listed: 4

#	Author Name	Gender	Nationality	Affiliation	Affiliation Theme
1st	Sophia Fürtjes	Female	Germany	TUD Dresden University of Technology, Behavioral Epidemiology, Institute of Clinical Psychology and Psychotherapy, Chemnitzer Straße 46, D-01187 Dresden, Germany	Clinical Psychology & Behavioral Science
2nd	Elisabeth Gebel	Female	Germany	TUD Dresden University of Technology, Behavioral Epidemiology, Institute of Clinical Psychology and Psychotherapy, Chemnitzer Straße 46, D-01187 Dresden, Germany	Clinical Psychology & Behavioral Science
3rd	Hanna Kische	Female	Germany	TUD Dresden University of Technology, Behavioral Epidemiology, Institute of Clinical Psychology and Psychotherapy, Chemnitzer Straße 46, D-01187 Dresden, Germany	Clinical Psychology & Behavioral Science

#	Author Name	Gender	Nationality	Affiliation	Affiliation Theme
Last	Katja Beesdo-Baum	Female	Germany	TUD Dresden University of Technology, Behavioral Epidemiology, Institute of Clinical Psychology and Psychotherapy, Chemnitzer Straße 46, D-01187 Dresden, Germany	Clinical Psychology & Behavioral Science

Galetsi et al., 2023

Authors listed: 4

#	Author Name	Gender	Nationality	Affiliation	Affiliation Theme
1st	Panagiota Galetsi	Female	Greece	School of Humanities, Social Sciences and Economics, International Hellenic University, Thessaloniki, Greece	Economics & Business
2nd	Sameer Kumar	Male	India	Opus College of Business, University of St. Thomas Minneapolis Campus, Minneapolis, Minnesota	Economics & Business
3rd	Korina Katsaliaki	Female	Greece	School of Humanities, Social Sciences and Economics, International Hellenic University, Thessaloniki, Greece	Economics & Business
Last	Mark Ferguson	Male	United States	Darla Moore School of Business, University of South Carolina, Columbia, South Carolina	Economics & Business

Hong et al., 2024

Authors listed: 5

#	Author Name	Gender	Nationality	Affiliation	Affiliation Theme
1st	Minji Hong	Female	South Korea	Department of Medical Device Engineering and Management, College of Medicine, Yonsei University, Seoul, Republic of Korea	Biomedical Engineering & Medical Devices
2nd	Vasuki Rajaguru	Male	South Korea	Department of Healthcare Management, Graduate School of Public Health, Yonsei University, Seoul, Republic of Korea	Health & Technology Management
3rd	KyungYi Kim	Female	South Korea	Department of Healthcare Management, Graduate School of Public Health, Yonsei University, Seoul, Republic of Korea	Health & Technology Management
4th	Suk-Yong Jang	Male	South Korea	Department of Healthcare Management, Graduate School of Public Health, Yonsei University, Seoul, Republic of Korea	Health & Technology Management

#	Author Name	Gender	Nationality	Affiliation	Affiliation Theme
Last	Sang Gyu Lee	Male	South Korea	Department of Preventive Medicine, College of Medicine, Yonsei University, Seoul, Republic of Korea	Public Health & Epidemiology

Jakob et al., 2024

Authors listed: 5 (source file recorded "Numb Authors" = 4 — see note)

#	Author Name	Gender	Nationality	Affiliation	Affiliation Theme
1st	Robert Jakob	Male	Germany	Center for Digital Health Interventions, Institute of Technology Management, University of St. Gallen, Dufourstrasse 40a, CH-9000 St. Gallen, Switzerland, Department of Management, Technology, and Economics, ETH Zurich	Health & Technology Management
2nd	Justas Narauskas	Female	Ireland	Center for Digital Health Interventions, Institute of Technology Management, University of St. Gallen, Dufourstrasse 40a, CH-9000 St. Gallen, Switzerland, Department of Management, Technology, and Economics, ETH Zurich	Health & Technology Management
3rd	Elgar Fleisch	Male	Austria	Center for Digital Health Interventions, Institute of Technology Management, University of St. Gallen, Dufourstrasse 40a, CH-9000 St. Gallen, Switzerland, Department of Management, Technology, and Economics, ETH Zurich	Health & Technology Management
4th	Laura Maria Konig	Female	Germany	Faculty of Life Sciences, University of Bayreuth, Kulmbach, Germany	Public Health & Epidemiology
Last	Tobias Kowatsch	Male	Switzerland	Center for Digital Health Interventions, Institute of Technology Management, University of St. Gallen, Dufourstrasse 40a, CH-9000 St. Gallen, Switzerland, Department of Management, Technology, and Economics, ETH Zurich	Health & Technology Management

Kozlov et al., 2020

Authors listed: 8 (source file recorded "Numb Authors" = 7 — see note)

#	Author Name	Gender	Nationality	Affiliation	Affiliation Theme
1st	Elissa Kozlov	Female	United States	Institute for Health, Health Policy and Aging Research, Rutgers University, New Brunswick, NJ, United States	Public Health & Epidemiology
2nd	Erin Bantum	Female	United States	Cancer Prevention in the Pacific, University of Hawaii Cancer Center, Honolulu, HI, United States	Public Health & Epidemiology
3rd	Ian Pagano	Male	United States	Cancer Prevention in the Pacific, University of Hawaii Cancer Center, Honolulu, HI, United States	Public Health & Epidemiology

#	Author Name	Gender	Nationality	Affiliation	Affiliation Theme
4th	Robyn Walser	Female	United States	National Center for PTSD Research, VA Palo Alto Healthcare System, Menlo Park, CA, United States	Clinical Psychology & Behavioral Science
5th	Kelly Ramsey	Female	United States	National Center for PTSD Research, VA Palo Alto Healthcare System, Menlo Park, CA, United States	Clinical Psychology & Behavioral Science
6th	Katherine Taylor	Female	United States	National Center for PTSD Research, VA Palo Alto Healthcare System, Menlo Park, CA, United States	Clinical Psychology & Behavioral Science
7th	Beth Jaworski	Female	United States	National Center for PTSD Research, VA Palo Alto Healthcare System, Menlo Park, CA, United States	Clinical Psychology & Behavioral Science
Last	Jason Owen	Male	United States	National Center for PTSD Research, VA Palo Alto Healthcare System, Menlo Park, CA, United States	Clinical Psychology & Behavioral Science

Lee et al., 2020

Authors listed: 5 (source file recorded "Numb Authors" = 4 — see note)

#	Author Name	Gender	Nationality	Affiliation	Affiliation Theme
1st	Jungeun Lee	Female	South Korea	Noom Inc, Seoul 07325, Korea.	Digital Health & Health Informatics
2nd	Sujin Bae	Male	South Korea	Office of Research, Chung-Ang University, Seoul 06974, Korea.	Economics & Business
3rd	Dohyung Park	Male	South Korea	Noom Inc, Seoul 07325, Korea.	Digital Health & Health Informatics
4th	Youngin Kim	Male	South Korea	Noom Inc, Seoul 07325, Korea.	Digital Health & Health Informatics
Last	Jisun Park	Female	South Korea	Department of Social Work and Counseling, Catholic University of Pusan, Pusan 46252, Korea.	Social Sciences & Humanities

Rasul et al., 2023

Authors listed: 4

#	Author Name	Gender	Nationality	Affiliation	Affiliation Theme
1st	Tareq Rasul	Male	Bangladesh	Gulf University for Science & Technology	Economics & Business
2nd	Aaron Wijeratne	Male	Australia	Australian Institute of Business	Economics & Business
3rd	Samaneh Soleimani	Female	Iran	Australian Institute of Business	Economics & Business
Last	Weng Marc Lim	Male	Australia	Sunway University	Economics & Business

Skeens et al., 2024

Authors listed: 4

#	Author Name	Gender	Nationality	Affiliation	Affiliation Theme
1st	Micah A Skeens	Female	United States	The Abigail Wexner Research Institute, Nationwide Children's Hospital, Columbus, OH, United States	Medicine & Clinical Practice
2nd	Daniel I Jackson	Male	United States	The Abigail Wexner Research Institute, Nationwide Children's Hospital, Columbus, OH, United States	Medicine & Clinical Practice
3rd	Malcolm S Sutherland-Foggio	Male	South Korea	The Abigail Wexner Research Institute, Nationwide Children's Hospital, Columbus, OH, United States	Medicine & Clinical Practice
Last	Emre Sezgin	Male	Turkey	The Abigail Wexner Research Institute, Nationwide Children's Hospital, Columbus, OH, United States	Medicine & Clinical Practice

Van der Haar et al., 2023

Authors listed: 4

#	Author Name	Gender	Nationality	Affiliation	Affiliation Theme
1st	Sandra van der Haar	Female	Netherlands	Wageningen Food & Biobased Research, Wageningen University & Research, Wageningen, Netherlands	Public Health & Epidemiology
2nd	Ireen Raaijmakers	Female	Netherlands	Wageningen Economic Research, Wageningen University & Research, Wageningen, Netherlands	Economics & Business
3rd	Muriel C D Verain	Female	Netherlands	Wageningen Economic Research, Wageningen University & Research, Wageningen, Netherlands	Economics & Business

#	Author Name	Gender	Nationality	Affiliation	Affiliation Theme
Last	Saskia Meijboom	Female	Netherlands	Wageningen Food & Biobased Research, Wageningen University & Research, Wageningen, Netherlands	Public Health & Epidemiology

Viswanathan et al., 2023

Authors listed: 3

#	Author Name	Gender	Nationality	Affiliation	Affiliation Theme
1st	Ambika V. Viswanathan	Female	United States	University of North Carolina School of Medicine, Chapel Hill, NC	Medicine & Clinical Practice
2nd	John A. Dodson	Male	United States	New York University Grossman School of Medicine, New York, NY	Medicine & Clinical Practice
Last	Nina L. Blachman	Female	United States	New York University Grossman School of Medicine, New York, NY	Medicine & Clinical Practice

Collins, 2025

Authors listed: 6

#	Author Name	Gender	Nationality	Affiliation	Affiliation Theme
1st	Rebecca A. Collins	Female	Australia	School of Health Sciences, College of Health Medicine and Wellbeing, The University of Newcastle, Callaghan, New South Wales, Australia Food and Nutrition Research Group, Hunter Medical Research Institute, New Lambton Heights, New South Wales, Australia	Public Health & Epidemiology
2nd	Melinda Hutchesson	Female	Australia	School of Health Sciences, College of Health Medicine and Wellbeing, The University of Newcastle, Callaghan, New South Wales, Australia Food and Nutrition Research Group, Hunter Medical Research Institute, New Lambton Heights, New South Wales, Australia	Public Health & Epidemiology
3rd	Marc Adam	Male	Australia	School of Information and Physical Sciences, College of Engineering, Science and Environment, The University of Newcastle, Callaghan, New South Wales, Australia	Digital Health & Health Informatics
4th	Erin D. Clarke	Female	Australia	School of Health Sciences, College of Health Medicine and Wellbeing, The University of Newcastle, Callaghan, New South Wales, Australia Food and Nutrition Research Group, Hunter Medical Research Institute, New Lambton Heights, New South Wales, Australia	Public Health & Epidemiology
5th	Clare E. Collins	Female	Australia	School of Health Sciences, College of Health Medicine and Wellbeing, The University of Newcastle, Callaghan, New South Wales, Australia Food and Nutrition Research Group, Hunter Medical Research Institute, New Lambton Heights, New South Wales, Australia	Public Health & Epidemiology

#	Author Name	Gender	Nationality	Affiliation	Affiliation Theme
Last	Tracy L. Burrows	Female	Australia	School of Health Sciences, College of Health Medicine and Wellbeing, The University of Newcastle, Callaghan, New South Wales, Australia Food and Nutrition Research Group, Hunter Medical Research Institute, New Lambton Heights, New South Wales, Australia	Public Health & Epidemiology§

Kanai, 2025

Authors listed: 9

#	Author Name	Gender	Nationality	Affiliation	Affiliation Theme
1st	Masashi Kanai	Male	Japan	PREVENT Inc., Aichi, Japan	Health & Technology Management
2nd	Yosuke Fujisawa	Male	Japan	SUMITOMO LIFE Insurance Company, Tokyo, Japan	Economics & Business
3rd	Takashi Hamatani	Male	Japan	SUMITOMO LIFE Insurance Company, Tokyo, Japan	Economics & Business
4th	Kakeru Tateyama	Male	Japan	SUMITOMO LIFE Insurance Company, Tokyo, Japan	Economics & Business
5th	Takahiro Miki	Male	Japan	PREVENT Inc., Aichi, Japan	Health & Technology Management
6th	Takuya Toda	Male	Japan	PREVENT Inc., Aichi, Japan	Health & Technology Management
7th	Tsutomu Sugawara	Male	Japan	PREVENT Inc., Aichi, Japan SUMITOMO LIFE Insurance Company, Tokyo, Japan	Health & Technology Management
8th	Yuta Hagiwara	Male	Japan	PREVENT Inc., Aichi, Japan	Health & Technology Management
Last	Naoki Onozato	Male	Japan	SUMITOMO LIFE Insurance Company, Tokyo, Japan	Economics & Business

Richards, 2025

Authors listed: 4

#	Author Name	Gender	Nationality	Affiliation	Affiliation Theme
1st	Rebecca Richards	Female	United Kingdom	Second Nature, London, United Kingdom	Health & Technology Management
2nd	Gina Wren	Female	United Kingdom	Nuffield Department of Primary Care Health Sciences, University of Oxford, Oxford, United Kingdom	—
3rd	Peta Campion	Female	United Kingdom	Second Nature, London, United Kingdom	Public Health & Epidemiology
Last	Michael Whitman	Male	United Kingdom	Second Nature, London, United Kingdom	Health & Technology Management

Fuente-Vidal, 2026

Authors listed: 8 (source file recorded "Numb Authors" = 7 — see note)

#	Author Name	Gender	Nationality	Affiliation	Affiliation Theme
1st	Andrea Fuente-Vidal	Female	Spain	Faculty of Psychology, Education and Sports Sciences (FPCEE) Blanquerna, Universitat Ramon Llull, Barcelona, Spain Research Group on Methodology, Methods, Models and Outcomes of Health and Social Sciences (M3O), Faculty of Health Sciences and Welfare, Centre for Health and Social Care Research (CESS), Universitat de Vic - Universitat Central de Catalunya, Vic, Spain	Clinical Psychology & Behavioral Science
2nd	Juan Manuel Arribas-Marin	Male	Spain	San Juan de Dios University School of Nursing and Physical Therapy, Comillas Pontifical University, Madrid, Spain San Juan de Dios Foundation, Madrid, Spain	Nursing & Health Care Sciences
3rd	Oscar Bastidas-Jossa	Male	Spain	eVIDA Research Group, Universidad de Deusto, Bilbao, Spain	Digital Health & Health Informatics
4th	Myriam Guerra-Balic	Female	Spain	Faculty of Psychology, Education and Sports Sciences (FPCEE) Blanquerna, Universitat Ramon Llull, Barcelona, Spain	Clinical Psychology & Behavioral Science
5th	Begonya Garcia-Zapirain	Female	Spain	eVIDA Research Group, Universidad de Deusto, Bilbao, Spain	Digital Health & Health Informatics
6th	Joel Montane	Male	Spain	Faculty of Psychology, Education and Sports Sciences (FPCEE) Blanquerna, Universitat Ramon Llull, Barcelona, Spain School of Health Sciences, Blanquerna, Universitat Ramon Llull, Barcelona, Spain	Clinical Psychology & Behavioral Science
7th	Javier Jerez-Roig	Male	Spain	Research Group on Methodology, Methods, Models and Outcomes of Health and Social Sciences (M3O), Faculty of Health Sciences and Welfare, Centre for Health and Social Care Research	Public Health & Epidemiology

#	Author Name	Gender	Nationality	Affiliation	Affiliation Theme
				(CESS), Universitat de Vic - Universitat Central de Catalunya, Vic, Spain Institute of Sport Science and Innovations, Lithuanian Sports University, Kaunas, Lithuania Institute for Research and Innovation in Life and Health Sciences in Central Catalonia (IRIS-CC), Universitat de Vic Universitat Central de Catalunya, Vic, Spain	
Last	Roger Prat	Male	Spain	Faculty of Psychology, Education and Sports Sciences (FPCEE) Blanquerna, Universitat Ramon Llull, Barcelona, Spain	Clinical Psychology & Behavioral Science
