# Supplementary material for: Beyond the app: a marketness assessment of digital health, sports, and well-being - a systematic review
Source: Front Digit Health. 2026 Oct 8;8:1871114. doi: 10.3389/fdgth.2026.1871114 (PMC13687406; doi:10.3389/fdgth.2026.1871114)
Supplement: Supplementary file 4 [file Datasheet4.pdf]

Supplementary Material A4: Systematic Review Data Extraction Framework

Marketness criterion	Variable	Operationalisation	Category scheme	Source
**Study descriptors**	Publication year	Year of publication	Numeric	—
	Country	Country of first author	Inductive	—
	Disciplinary background	Primary discipline of author team inferred from affiliations	Biomedical engineering / health informatics / health technology management; Psychology and behavioural sciences; Clinical or public health; Economics or business; Other	(1)
	Study design	Empirical design class for MMAT alignment	Qualitative; Quantitative descriptive; Quantitative non-randomised; Mixed methods	(2)
	Study context	Relationship of study to solution	Market study; Solution evaluation	—

	Developer type	Organisational anchoring of solution developer	Private-sector; Public or non-profit; Cross-sector collaboration	(3,4,5)
	Value proposition cluster	Cluster assignment based on coded value proposition and application context	Condition management; Lifestyle and behaviour; Physical activity	Inductive
**Typified goods or services**	Functional purpose	Core purpose coded using WHO Classification of Digital Health Interventions	WHO DHI categories	(6)
	Intervention components	Delivery logic structured using BIT model	BIT model components	(7)
	Behaviour-cha nge features	Persuasive and behaviour-change features	Self-monitoring; Feedback; Prompts/reminders; Goal setting/personalisation; Social support/connectivity	Inductive
	Access and pricing model	User-facing price structure determining terms of exchange	Free; Freemium; Paid subscription; Incentive-linked; Advertising-supported	—

**Voluntary and specified exchange**	Agent types	Agents identified and their roles in the exchange structure	Primary agents; Secondary agents; Professional agents; Solution developers; Distribution platforms; Institutional bodies; Peer and community networks	(3,8)
	Voluntarity of participation	Whether participation was freely initiated or externally constrained	Voluntary; Constrained by clinical protocol; Constrained by referral pathway; Constrained by institutional mandate	—
	Exchange terms	How terms of exchange were defined	Freely negotiated; Defined by app onboarding; Governed by clinical authority; Governed by regulatory authority	—
**Regularity and competition**	Entry drivers	Factors shaping adoption decisions	Endorsement and legitimacy; Perceived value and usefulness; Pricing and access model	(9,10,11,12)
	Retention drivers	Factors sustaining continued engagement	Perceived value and usefulness; Supportive design features; Financial incentives	(7,13)
	Exit drivers	Factors contributing to discontinuation	Information quality or credibility concerns; Logging burden or time investment; Loss of interest or relevance; Privacy or data concerns; Cost concerns	(9,14)
	Competitive dynamics	Evidence of competition among solutions	Agent switching; Price pressure among developers; Developer pricing responses; None reported	—
**Interactions and coordination mechanisms**	Agent–agent ties	Relationship types among agents	Authority/power; Instrumental; Communication; Boundary-penetration; Transaction	(15)
	Agent–solution ties	Relationship types between agents and digital solutions	Authority/power; Instrumental; Communication; Boundary-penetration; Transaction	(15)

References:

Choi BCK, Pak AWP. Clin Investig Med. 2006;29(6):351–64.

Hong QN et al. Educ Inf. 2018;34(4):285–91.

van Gemert-Pijnen JE et al. J Med Internet Res. 2011;13(4):e111.

Triberti S, Riva G. Patient Engagem Consum Centered Model Innov Healthc. 2015;56–65.

Boonstra A et al. BMC Health Serv Res. 2014;14(1):370.

World Health Organization. Classification of digital health interventions v1.0. 2018.

Mohr DC et al. J Med Internet Res. 2014;16(6):e146.

Rochet JC, Tirole J. J Eur Econ Assoc. 2003;1(4):990–1029.

Greenhalgh T et al. J Med Internet Res. 2017;19(11):e8775.

Davis FD. MIS Q. 1989;13(3):319.

Venkatesh V et al. MIS Q. 2012;36(1):157–78.

Drummond M. Methods for the economic evaluation of health care programmes. 2015.

Michie S et al. J Med Internet Res. 2017;19(6):e232.

Sunstein CR. Behav Public Policy. 2022;6(4):654–73.

Thompson G et al. Markets, hierarchies, and networks. Sage Publications; 1991.
