# Supplementary material for: Beyond the app: a marketness assessment of digital health, sports, and well-being - a systematic review
Source: Front Digit Health. 2026 Oct 8;8:1871114. doi: 10.3389/fdgth.2026.1871114 (PMC13687406; doi:10.3389/fdgth.2026.1871114)
Supplement: Supplementary file 5 [file Datasheet5.pdf]

Category of Study Desgin	Criterion	Chahal et al., 2021	Chen et al., 2024	Farič et al., 2021	Fürtjes et al., 2024	Galetsi et al., 2023	Hong et al., 2024	Jakob et al., 2024	Kozlov et al., 2020	Lee et al., 2020	Rasul et al., 2023	Skeens et al., 2024	Van der Haar et al., 2023	Viswanathan et al., 2023	Collins, 2025	Kanai, 2025	Richards, 2025	Fuente-Vidal et al., 2026
Screening questions	Are there clear research questions?	Y	Y	Y	Y	Y	Y	Y	Y	Y	Y	Y	Y	Y	Y	Y	Y	Y
	Do the collected data allow to address the research questions?	Y	Y	Y	Y	Y	Y	Y	Y	Y	Y	Y	Y	Y	Y	Y	Y	Y
Qualitative Studies	Is the qualitative approach appropriate to answer the research question?			Y														
	Are the qualitative data collection methods adequate to address the research question?			Y														
	Are the findings adequately derived from the data?			Y														
	Is the interpretation of results sufficiently substantiated by data?			Y														
	Is there coherence between qualitative data sources, collection, analysis and interpretation?			Y														
Quantitative Randomized Controlled Trials	Is randomization appropriately performed?																	
	Are the groups comparable at baseline?																	
	Are there complete outcome data?																	
	Are outcome assessors blinded to the intervention provided?																	
	Did the participants adhere to the assigned intervention?																	
Quantitative Non-randomized Studies	Are the participants representative of the target population?							Y		N					N	N	N	
	Are measurements appropriate regarding both the outcome and intervention (or exposure)?							Y		Y					Y	Y	Y	
	Are there complete outcome data?							Y		N					N	Y	N	
	Are the confounders accounted for in the design and analysis?							Y		Y					Y	Y	N	
	During the study period, is the intervention administered (or exposure occurred) as intended?							Y		Y					Y	Y	Y	
Quantitative Descriptive Studies	Is the sampling strategy relevant to address the research question?	Y	Y		N	Y			Y		Y	Y		Y				Y
	Is the sample representative of the target population?	N	N		N	CT			CT		CT	Y		CT				CT
	Are the measurements appropriate?	Y	Y		Y	Y			Y		Y	Y		Y				Y
	Is the risk of nonresponse bias low?	CT	Y		N	Y			CT		CT	CT		CT				CT
	Is the statistical analysis appropriate to answer the research question?	Y	Y		Y	Y			Y		Y	Y		N				Y
Mixed Methods Studies	Is there an adequate rationale for using a mixed methods design to address the research question?						Y						Y					
	Are the different components of the study effectively integrated to answer the research question?						Y						Y					
	Are the outputs of the integration of qualitative and quantitative components adequately interpreted?						Y						Y					
	Are divergences and inconsistencies between qualitative and quantitative results adequately addressed?						CT						N					
	Do the different components of the study adhere to the quality criteria of each tradition of the methods involved?						Y						CT					
