# Supplementary material for: Beyond the app: a marketness assessment of digital health, sports, and well-being - a systematic review
Source: Front Digit Health. 2026 Oct 8;8:1871114. doi: 10.3389/fdgth.2026.1871114 (PMC13687406; doi:10.3389/fdgth.2026.1871114)
Supplement: Supplementary file 6 [file Datasheet6.pdf]

Supplementary Material A6: Individual Study Data

Table 1: Study descriptors

Author, year	Publication year	Country (first author)	Disciplinary background	Study design	Study context	Developer type	Value proposition
**Chahal et al., 2021**	2021	Canada	Economics or business	Quantitative descriptive	Market study	Private-sector	Condition management – Symptom management and care support for RA
**Chen et al., 2024**	2024	Taiwan	Clinical or public health	Quantitative descriptive	Market study	Private-sector	Condition management – Cognitive support and monitoring for dementia
**Galetsi et al., 2023**	2023	Greece	Economics or business	Quantitative descriptive	Market study	Private-sector	Condition management – Diagnostic and educational tools for health professionals
**Skeens et al., 2024**	2024	United States	Clinical or public health	Quantitative descriptive	Market study	Public or non-profit	Condition management – Care coordination and coping support in paediatric cancer
**Viswanathan et al., 2023**	2023	United States	Clinical or public health	Quantitative descriptive	Solution evaluation	Cross-sector collaboration	Condition management – Geriatric assessment tools for clinical training
**Fürtjes et al., 2024**	2024	Germany	Psychology and behavioural sciences	Quantitative descriptive	Market study	Private-sector	Lifestyle and behaviour – Self-guided mental well-being support
**Hong et al., 2024**	2024	Republic of Korea	Clinical or public health	Mixed methods	Market study	Private-sector	Lifestyle and behaviour – Menstrual cycle tracking and self-understanding
**Jakob et al., 2024**	2024	Switzerland	Biomedical engineering / health informatics / health technology management	Quantitative non-randomised	Solution evaluation	Cross-sector collaboration	Lifestyle and behaviour – Dietary self-regulation and nutrition literacy
**Kozlov et al., 2020**	2020	United States	Clinical or public health	Quantitative non-randomised	Solution evaluation	Public or non-profit	Lifestyle and behaviour – Mindfulness training for psychological well-being
**Lee et al., 2020**	2020	South Korea	Clinical or public health	Quantitative non-randomised	Market study	Private-sector	Lifestyle and behaviour – Weight management through financial incentives
**Rasul et al., 2023**	2023	Not reported (author affiliations: Kuwait, Australia)	Economics or business	Quantitative descriptive	Market study	Private-sector	Lifestyle and behaviour – General health self-management

Author, year	Publication year	Country (first author)	Disciplinary background	Study design	Study context	Developer type	Value proposition
**van der Haar et al., 2023**	2023	Netherlands	Economics or business	Mixed methods	Market study	Private-sector	Lifestyle and behaviour – Nutrition self-tracking and dietary behaviour change
**Farič et al., 2021**	2021	United Kingdom	Psychology and behavioural sciences	Qualitative	Market study	Private-sector	Physical activity – Physical activity through narrative gamification
**Collins, 2025**	2025	Australia	Clinical or public health	Quantitative non-randomised	Market study	Public or non-profit	Lifestyle and behaviour – Diet quality improvement through financial incentivisation
**Kanai, 2025**	2025	Japan	Biomedical engineering / health informatics / health technology management	Quantitative non-randomised	Market study	Private-sector	Physical activity – Physical activity through insurance-based incentives
**Richards, 2025**	2025	United Kingdom	Biomedical engineering / health informatics / health technology management	Quantitative non-randomised	Market study	Private-sector	Condition management – Weight management through GLP-1RA medication and digital coaching
**Fuente-Vidal et al., 2026**	2026	Spain	Psychology and behavioural sciences	Quantitative descriptive	Market study	Private-sector	Physical activity – Fitness app engagement and training behaviour

Table 2: Typified goods or services

Author, year	Functional purpose	Behaviour-change features	Access and pricing model
**Chahal et al., 2021**	Client health records / self-monitoring; provider-to-client communication (reminders, scheduling)	Self-monitoring; Prompts/reminders	Freemium
**Chen et al., 2024**	Client-facing decision support and self-management (cognitive training games, dementia education)	Self-monitoring; Goal setting/personalisation (cognitive training games)	Free (majority; app review sample downloadable without cost)
**Galetsi et al., 2023**	Provider-facing decision support (diagnostic aid) and educational content	Feedback (app quality/ratings feedback loop implied); prompts not centrally examined	Advertising-supported
**Skeens et al., 2024**	Client-facing self-management and care coordination (resource sharing, symptom tracking, care team connection, journaling, community support)	Self-monitoring; Social support/connectivity (community support, care team connection); Prompts/reminders (appointment tracking)	Predominantly free (majority publicly accessible at no cost); some apps freemium with in-app purchases up to US $60
**Viswanathan et al., 2023**	Provider/trainee-facing decision support and educational tool (comprehensive geriatric assessment across 8 domains)	Self-monitoring (assessment tracking); Feedback (results and next-steps guidance)	Free (cost-free access, described as the first free comprehensive geriatric assessment app)
**Fürtjes et al., 2024**	Client-facing self-help and self-care content (mindfulness, mood-tracking, relaxation)	Self-monitoring (mood tracking)	Freemium
**Hong et al., 2024**	Client-facing self-monitoring and prediction (cycle prediction, symptom/mood logging)	Self-monitoring; Prompts/reminders	Freemium
**Jakob et al., 2024**	Client-facing self-monitoring and behaviour-change support (dietary self-monitoring via conversational agent, nutrition literacy)	Self-monitoring (food diary); Prompts/reminders (follow-up reminders); Goal setting/personalisation (choice of conversational agent persona)	Free and publicly available (no monetary incentives)
**Kozlov et al., 2020**	Client-facing self-guided training (mindfulness course: training plan, practice exercises, learning topics, tracking)	Self-monitoring (tracking); Prompts/reminders (push notifications, optional)	Free (publicly available at no cost)
**Lee et al., 2020**	Client-facing self-monitoring and behaviour-change support (food logging, physical activity tracking) with financial reimbursement mechanism	Self-monitoring (food/weight/activity logging); Goal setting/personalisation	Paid subscription with incentive-linked reimbursement (participants purchased a 16-week programme; received monetary reimbursement up to full amount paid, contingent on logging adherence)
**Rasul et al., 2023**	Client-facing self-management support (sugar/diet-related tracking and information, general m-Health functionality)	Not specifically coded (study models continuance intention via general technology-acceptance constructs, not discrete BCT features)	Freemium
**van der Haar et al., 2023**	Client-facing self-monitoring and behaviour-change support (food tracking, goal setting, feedback across start/use/end phases)	Self-monitoring (food tracking); Feedback; Prompts/reminders; Goal setting/personalisation	Freemium
**Farič et al., 2021**	Client-facing engagement/motivation tool (narrative-driven exergame layered on running/cycling/walking activity)	Self-monitoring (implied via activity tracking during runs); Goal setting/personalisation (narrative-driven pacing); Social support/connectivity (peer community referenced by users)	Freemium (free trial tier plus paid subscription/in-app purchase options)
**Collins, 2025**	Client-facing self-management and behaviour-change support (Healthy Eating Quiz, personalised nutrition report, weekly nutrition-challenge emails)	Self-monitoring (diet quality quiz); Prompts/reminders (weekly emails); Goal setting/personalisation (personalised report); Financial incentive (prize draws for engaged/completing participants)	Free access (publicly available website and challenge), with optional financial incentive (prize draws of AUD$25–100 gift cards)
**Kanai, 2025**	Client-facing self-monitoring and behaviour-change support (wearable/app step tracking) linked to an insurance premium incentive mechanism	Self-monitoring (step and heart-rate tracking); Goal setting/personalisation (weekly step targets); Financial incentive (points-based system reducing insurance premiums; tangible rewards for meeting targets)	Insurance-linked incentive model (points accumulate toward premium discounts and tiered benefits; embedded within a paid insurance product, not a standalone free/paid app)

Author, year	Functional purpose	Behaviour-change features	Access and pricing model
**Richards, 2025**	Client-facing behaviour-change support and medication-assisted weight management (health-coach messaging, connected-scale self-monitoring, educational content)	Self-monitoring (weight via connected scales); Feedback (one-to-one health-coach messaging); Goal setting/personalisation (individualised weight-loss, step, and sleep goals); Prompts/reminders (engagement and check-in notifications)	Paid subscription (£179–299/month, varying with medication dose)
**Fuente-Vidal et al., 2026**	Client-facing self-monitoring and training-engagement support (personalised training routines, workout tracking)	Self-monitoring (training frequency/adherence tracking); Goal setting/personalisation (fitness goals, training schedule)	Freemium (free tier plus paid "Pro version" subscription)

Table 3: Voluntary and specified exchange

Author, year	Agent types	Voluntarity of participation	Exchange terms
**Chahal et al., 2021**	Primary agents (patients); Professional agents (physicians recommending apps); Solution developers	Constrained by referral pathway (physician recommendation pathway reported)	Freely negotiated / defined by commercial app pricing model
**Chen et al., 2024**	Primary agents (patients); Secondary agents (caregivers); Solution developers; Distribution platforms (App Store/Google Play)	Voluntary	Defined by app onboarding / app-store listing
**Galetsi et al., 2023**	Primary agents (health professionals/medical students as app users); Solution developers; Distribution platforms	Voluntary	Defined by app onboarding / commercial app-store pricing model
**Skeens et al., 2024**	Primary agents (patients); Secondary agents (caregivers); Professional agents (care team connection feature); Solution developers; Distribution platforms	Voluntary	Defined by app onboarding / app-store listing
**Viswanathan et al., 2023**	Primary agents (trainees and clinicians); Institutional bodies (developed with academic/clinical input; instrument creators consulted); Solution developers	Constrained by clinical protocol (institutional/clinical training context)	Defined by app onboarding
**Fürtjes et al., 2024**	Primary agents (general population users); Professional agents (prescribers, for the small prescription-based DiGA subset); Institutional bodies (statutory health insurance for DiGA); Solution developers; Distribution platforms	Voluntary	Defined by app onboarding (general use); governed by regulatory authority for prescription-based DiGA subset
**Hong et al., 2024**	Primary agents (general population, millennial/Gen Z women); Solution developers; Distribution platforms	Voluntary	Defined by app onboarding
**Jakob et al., 2024**	Primary agents (general population users); Solution developers; Distribution platforms (app-store/onboarding channels: personal referrals, clinical referrals, app-store discovery)	Voluntary	Defined by app onboarding
**Kozlov et al., 2020**	Primary agents (general population users); Institutional bodies (VA-developed protocols underpin app content); Solution developers; Distribution platforms	Voluntary (assessment/tutorial recommended but not required)	Defined by app onboarding
**Lee et al., 2020**	Primary agents (participants); Solution developers (Noom)	Voluntary	Freely negotiated (commercial purchase) with incentive-linked reimbursement terms defined by app programme
**Rasul et al., 2023**	Primary agents (general population m-Health users); Solution developers	Voluntary	Not reported
**van der Haar et al., 2023**	Primary agents (current/former users and non-users); Solution developers; Distribution platforms	Voluntary	Defined by app onboarding
**Farič et al., 2021**	Primary agents (runners/general population users); Peer and community networks (online ZR community); Solution developers (Six to Start)	Voluntary	Freely negotiated (freemium/subscription structure)
**Collins, 2025**	Primary agents (adult participants); Solution developers (NMNT platform team)	Voluntary	Free access defined by challenge enrolment terms; prize-draw incentive terms set by programme, not a priced exchange
**Kanai, 2025**	Primary agents (insurance policyholders); Institutional bodies (SUMITOMO LIFE Insurance; Discovery Ltd., programme designer); Solution developers (PREVENT Inc.)	Constrained by insurance contract (participation embedded within an existing policy, not a standalone opt-in)	Defined by insurance policy contract; premium adjustments tied to points accumulated through activity
**Richards, 2025**	Primary agents (patients); Professional agents (prescribing pharmacists, health coaches); Solution developers (Second Nature)	Voluntary (self-referral via digital marketing, word-of-mouth, or search)	Freely negotiated (commercial subscription pricing, £179–299/month depending on medication dose)

Author, year	Agent types	Voluntarity of participation	Exchange terms
**Fuente-Vidal et al., 2026**	Primary agents (general population app users); Solution developers; Distribution platforms	Voluntary	Defined by app onboarding / freemium structure with paid "Pro version" subscription tier

Table 4: Regularity and competition

Author, year	Entry drivers	Retention drivers	Exit drivers	Competitive dynamics
**Chahal et al., 2021**	Endorsement and legitimacy (physician recommendation significantly associated with adoption); Perceived value and usefulness (symptom tracking, scheduling, reminders preferred)	Not reported (study addresses adoption and WTP, not continued use)	Not reported	None reported
**Chen et al., 2024**	Perceived value and usefulness (app quality positively correlated with downloads)	Not reported	Not reported	None reported
**Galetsi et al., 2023**	Perceived value and usefulness (app quality, usefulness ratings positively associated with downloads)	Not directly examined (cross-sectional design; no longitudinal retention data)	Not reported	None reported (study notes market volume/downloads but not agent switching)
**Skeens et al., 2024**	Perceived value and usefulness (feature availability, e.g. resource sharing, symptom tracking)	Not reported (systematic market analysis, not a longitudinal usage study)	Not reported	None reported (authors note gap in apps for non-English-speaking/alternative-language markets, but no agent switching or price competition documented)
**Viswanathan et al., 2023**	Perceived value and usefulness (addressing market gap of paywalled/fragmented tools); Endorsement and legitimacy (instrument creators consulted, e.g. Mini-Cog co-creator)	Not reported (market survey and prototype description, no longitudinal use data)	Not reported	None reported
**Fürtjes et al., 2024**	Perceived value and usefulness (helpfulness, fun, availability cited as top reasons for use)	Not reported (cross-sectional, no continued-use data)	Information quality or credibility concerns (distrust); Privacy or data concerns (data security cited as barrier by non-users)	None reported
**Hong et al., 2024**	Perceived value and usefulness (cycle prediction utility)	Not directly measured (cross-sectional survey plus qualitative interviews, not longitudinal retention)	Information quality or credibility concerns (app prediction inaccuracy cited as reason for quitting in qualitative interviews)	None reported
**Jakob et al., 2024**	Perceived value and usefulness; onboarding channel effects (daytime/weekday installation associated with better adherence)	Supportive design features (tutorials, customisable reminders, tailored conversational agent persona associated with adherence/long-term adherence)	Logging burden or time investment (implied by high early discontinuation/churn); users skipping tutorial less adherent	Authors note inability to assess competitive impact of other nutrition apps as a limitation – not empirically examined
**Kozlov et al., 2020**	Not directly examined as adoption driver (study is descriptive of reach/use, not adoption motivations)	Not directly examined; retention and rolling-retention measured descriptively but not linked to specific design features	Not reported	None reported
**Lee et al., 2020**	Perceived value and usefulness; Pricing and access model (deposit-contract/reimbursement structure explicitly designed to drive entry and engagement)	Financial incentives (monetary reimbursement for consistent logging, associated with continued engagement) – only reported case of a financial retention mechanism across all included studies	Not reported (retrospective analysis of successful vs unsuccessful loggers, not exit reasons)	None reported
**Rasul et al., 2023**	Perceived value and usefulness (ease of use, perceived usefulness modelled as continuance-intention antecedents)	Not clearly distinguished from entry drivers in this model-testing study	Implied by low continuance intention scores but not itemised into discrete exit reasons	None reported
**van der Haar et al., 2023**	Perceived value and usefulness (clear introduction/purpose, flexible tracking cited as top starting-phase drivers)	Supportive design features (database reliability, user-friendliness, continuously offered new information cited as top use/end-phase retention drivers)	Logging burden or time investment (high time investment was the most frequently cited reason for quitting, 37%)	None reported

Author, year	Entry drivers	Retention drivers	Exit drivers	Competitive dynamics
**Farič et al., 2021**	Perceived value and usefulness (narrative appeal, immersion); Pricing and access model (free trial lowering entry barrier)	Supportive design features (narrative/story engagement, community); no financial retention mechanism reported	Cost concerns (purchase of membership cited as an initial barrier for some); Loss of interest or relevance (one user reported stopping use)	None reported
**Collins, 2025**	Not reported (motivators for completing the Healthy Eating Quiz were recorded — e.g., "to know more about how to eat better," "to achieve or maintain a healthy weight" — but not analysed as adoption/entry drivers of the solution itself)	Financial incentives (RE$ET, the incentivised challenge, had significantly higher 6-week retention than the non-incentivised Spring Eatz challenge: 21% vs 16%, $z=2.14$, $p<0.05$)	Not reported	Not reported
**Kanai, 2025**	Not reported	Financial incentives (ongoing points-based premium discounts and tangible rewards were associated with a sustained increase in average daily step count over 36 months, from 7239.7 to 9392.7 steps/day, $p<0.001$)	Not reported (study population restricted to participants with 3 years of uninterrupted participation; those who discontinued were excluded, and reasons for discontinuation were not examined)	None reported
**Richards, 2025**	Not reported (recruitment channels are described — digital marketing, word-of-mouth referrals, search engine results — but not analysed as adoption drivers)	Not identified (no statistically significant differences in baseline characteristics were found between participants who remained active and those who became inactive at 12 months, $P>.05$ for all comparisons)	Reported directly: financial/cost (n=82), side effects (n=54), personal (n=22), lack of progress (n=17), goal achieved (n=15), service issues (n=5), other (n=4), no reason given (n=7)	None reported
**Fuente-Vidal et al., 2026**	None reported	Reported directly: intrinsic motivation was significantly associated with retention ($r=0.19$, $p=.01$); identified extrinsic motivation was also significantly associated with retention ($p<.05$); introjected extrinsic motivation was not significantly associated	Not reported	None reported

Table 5: Interactions and coordination mechanisms

Author, year	Agent–agent ties	Agent–solution ties
**Chahal et al., 2021**	Instrumental (physician recommends app to patient)	Instrumental (symptom tracking, scheduling); Transaction (payment for subscription/features)
**Chen et al., 2024**	Not reported (no agent–agent interaction examined; expert reviewers assessed apps, not patients/caregivers)	Instrumental (cognitive training, information provision)
**Galetsi et al., 2023**	Not reported (professional adoption behaviour modelled via TAM/DOI constructs, not direct agent–agent ties)	Instrumental (diagnostic/educational use); Transaction (in-app purchases, advertising exposure)
**Skeens et al., 2024**	Instrumental (care team connection feature; not deeply characterised)	Instrumental (symptom tracking, journaling, resource access)
**Viswanathan et al., 2023**	Authority/power (instrument creators validating tool content)	Instrumental (assessment administration, education/next-steps guidance)
**Fürtjes et al., 2024**	Not reported	Instrumental (mood tracking, mindfulness exercises)
**Hong et al., 2024**	Not reported	Instrumental (cycle prediction, symptom logging)
**Jakob et al., 2024**	Not reported	Instrumental (food diary logging); Communication (conversational agent interaction)
**Kozlov et al., 2020**	Not reported	Instrumental (mindfulness training exercises, tracking)
**Lee et al., 2020**	Not reported	Instrumental (logging); Transaction (purchase and reimbursement)
**Rasul et al., 2023**	Not reported	Instrumental (general app interaction, not solution-specific)
**van der Haar et al., 2023**	Not reported	Instrumental (food tracking, goal setting); Communication (app-provided feedback and information)
**Farič et al., 2021**	Instrumental / Communication (peer community interaction referenced but not structurally characterised)	Instrumental (narrative-driven exercise engagement); Transaction (in-app purchases)
**Collins, 2025**	Not reported	Instrumental (Healthy Eating Quiz completion, personalised report); Communication (weekly email content); Transaction (prize-draw eligibility contingent on engagement)
**Kanai, 2025**	Not reported	Instrumental (step and heart-rate tracking via wearable/app); Transaction (points accumulation tied to insurance premium adjustments)
**Richards, 2025**	Instrumental (health-coach messaging and one-to-one support; prescribing pharmacist oversight of medication)	Instrumental (weight tracking via connected scales, medication toolbox); Communication (asynchronous messaging with health coach, moderated peer support groups); Transaction (paid subscription, £179–299/month)
**Fuente-Vidal et al., 2026**	Not reported	Instrumental (training tracking, workout logging); Transaction (Pro version subscription)
